# Casein kinase 2 complex: a central regulator of multiple pathobiological signaling pathways in *Cryptococcus neoformans*

**DOI:** 10.1128/mbio.03275-23

**Published:** 2024-01-09

**Authors:** Yeseul Choi, Seong-Ryong Yu, Yujin Lee, Ann-Yae Na, Sangkyu Lee, Joseph Heitman, Ran Seo, Han-Seung Lee, Jong-Seung Lee, Yong-Sun Bahn

**Affiliations:** 1 Department of Biotechnology, College of Life Science and Biotechnology, Yonsei University, Seoul, South Korea; 2 School of Pharmacy, Sungkyunkwan University, Suwon, South Korea; 3 Department of Molecular Genetics and Microbiology, Duke University Medical Center, Durham, North Carolina, USA; 4 AmtixBio Co., Ltd., Hanam-si, Gyeonggi-do, South Korea; Universidade de Sao Paulo, Ribeirao Preto, Sao Paulo, Brazil

**Keywords:** CK2, Cka1, Ckb1, Ckb2, fungal meningitis

## Abstract

The casein kinase 2 (CK2) complex has garnered extensive attention over the past decades as a potential therapeutic target for diverse human diseases, including cancer, diabetes, and obesity, due to its pivotal roles in eukaryotic growth, differentiation, and metabolic homeostasis. While CK2 is also considered a promising antifungal target, its role in fungal pathogens remains unexplored. In this study, we investigated the functions and regulatory mechanisms of the CK2 complex in *Cryptococcus neoformans*, a major cause of fungal meningitis. The cryptococcal CK2 complex consists of a single catalytic subunit, Cka1, and two regulatory subunits, Ckb1 and Ckb2. Our findings show that Cka1 plays a primary role as a protein kinase, while Ckb1 and Ckb2 have major and minor regulatory functions, respectively, in growth, cell cycle control, morphogenesis, stress response, antifungal drug resistance, and virulence factor production. Interestingly, triple mutants lacking all three subunits (*cka1*Δ *ckb1*Δ *ckb2*Δ) exhibited more severe phenotypic defects than the *cka1*Δ mutant alone, suggesting that Ckb1/2 may have Cka1-independent functions. In a murine model of systemic cryptococcosis, *cka1*Δ and *cka1*Δ *ckb1*Δ *ckb2*Δ mutants showed severely reduced virulence. Transcriptomic, proteomic, and phosphoproteomic analyses further revealed that the CK2 complex controls a wide array of effector proteins involved in transcriptional regulation, cell cycle control, nutrient metabolisms, and stress responses. Most notably, CK2 disruption led to dysregulation of key signaling cascades central to *C. neoformans* pathogenicity, including the Hog1, Mpk1 MAPKs, cAMP/PKA, and calcium/calcineurin signaling pathways. In summary, our study provides novel insights into the multifaceted roles of the fungal CK2 complex and presents a compelling case for targeting it in the development of new antifungal drugs.

**IMPORTANCE:**

The casein kinase 2 (CK2) complex, crucial for eukaryotic growth, differentiation, and metabolic regulation, presents a promising therapeutic target for various human diseases, including cancer, diabetes, and obesity. Its potential as an antifungal target is further highlighted in this study, which explores CK2’s functions in *C. neoformans*, a key fungal meningitis pathogen. The CK2 complex in *C. neoformans*, comprising the Cka1 catalytic subunit and Ckb1/2 regulatory subunits, is integral to processes like growth, cell cycle, morphogenesis, stress response, drug resistance, and virulence. Our findings of CK2’s role in regulating critical signaling pathways, including Hog1, Mpk1 MAPKs, cAMP/PKA, and calcium/calcineurin, underscore its importance in *C. neoformans* pathogenicity. This study provides valuable insights into the fungal CK2 complex, reinforcing its potential as a target for novel antifungal drug development and pointing out a promising direction for creating new antifungal agents.

## INTRODUCTION

The casein kinase 2 (CK2) complex is an evolutionarily conserved entity that performs a wide range of cellular functions in eukaryotes, including cell cycle control, morphogenesis, differentiation, stress responses, and metabolism (1, 2). Over the past several decades, substantial research has been dedicated to understanding both the functional and structural aspects of CK2 (3). Unlike many other kinases, which often have a specific target substrate within a signaling or metabolic pathway, CK2 is known for its broad substrate specificity, phosphorylating hundreds of proteins (4, 5). This diversity underscores its multifaceted roles in biological processes. As a result, CK2 has gained prominence as a therapeutic target for various human diseases, ranging from cancer to neurological disorders, inflammation, autoimmune diseases, diabetes, and obesity (6). Recently, it has been reported that CK2 activity is induced in host cells upon SARS-CoV-2 infection, which is responsible for the COVID-19 pandemic, further highlighting the complex’s critical role in cellular processes (7).

The CK2 complex in eukaryotes typically has a tetrameric structure composed of two catalytic subunits and two regulatory subunits. In humans, the catalytic subunits are CK2α and CK2α′, and they exhibit a high sequence identity of 90% (8). Despite their high sequence identity, the carboxy-terminal domains of CK2α and CK2α′ are distinct and responsible for their functional differences (8, 9). Recently, additional isoforms of catalytic subunits have been discovered, likely due to alternative splicing, but their effects on CK2 function remain unclear (10). The regulatory subunit of the mammalian CK2 complex is CK2β, which interacts with substrates and stabilizes the holoenzyme (11). CK2β can be autophosphorylated by the catalytic subunits and forms a dimer *via* its middle domain, interacting with either CK2α or CK2α′ using its C-terminus (3, 12).

CK2 is widely expressed in human tissues and regulates cell survival, transcription, translation, and cell cycle progress (1, 13, 14). It plays a crucial role in governing cell cycle transitions, including G_0_/G_1_, G_1_/S, and G_2_/M (15, 16). Overexpression of CK2α can induce tumors, while inhibition of CK2 activity can lead to apoptosis through impacts on TNF-α, TRAIL, and FasL (17, 18). CK2α has been explored as an anti-cancer therapeutic target, with CX-4945 being a specific inhibitor that can induce apoptosis in cancer cells (19). Recently, CX-4945 has been designated by the U.S. FDA as an orphan drug for the treatment of cholangiocarcinoma (20).

The CK2 complex in fungi has been most extensively characterized in the model budding yeast, *Saccharomyces cerevisiae*. In this organism, the CK2 complex comprises two catalytic subunits (Cka1 and Cka2) and two regulatory subunits (Ckb1 and Ckb2). The yeast CK2 plays a pivotal role in regulating cell morphology and polarity by controlling microtubule assembly and dynamics (21). It interacts with the Arp2/3 complex by binding to Cmd1, affecting endocytosis and cell cycle progression by controlling the actin and tubulin cytoskeletons, respectively (22). Furthermore, CK2 phosphorylates Cdc28 at Ser46, and site-specific mutation of this residue reduces cell volume (23). Similarly, mutation of the CK2 phosphorylation site of Sic1, which is involved in controlling cell cycle and size, can cause dysregulated yeast cell size and volume (24).

The role of the CK2 complex in fungal pathogens has been partially characterized in *Candida albicans*. Similar to *S. cerevisiae*, the *C. albicans* CK2 comprises two catalytic subunits (Cka1 and Cka2) and two regulatory subunits (Ckb1 and Ckb2). CK2 plays a critical role in regulating the phosphorylation status of Hog1 by phosphorylating Hsp90, a crucial molecular chaperone, and controlling its protein levels (25). Interestingly, the regulatory subunits of CK2, rather than the catalytic subunits, play a significant role in Hsp90 regulation, indicating that each subunit of CK2 has distinct functions in this fungal pathogen. Reflecting the structural similarity among CK2 catalytic subunits, CX-4945 also inhibits the fungal CK2 catalytic subunits in *S. cerevisiae* and *C. albicans* (26). CX-4945 can prevent *C. albicans* from undergoing hyphal growth and adhesion. However, the minimum inhibitory concentration to kill the fungal species is higher than in mammals, suggesting that a fungal-specific CK2 inhibitor should be developed as a potential antifungal drug.

*Cryptococcus neoformans* is an opportunistic human fungal pathogen responsible for more than 180,000 deaths annually worldwide (27, 28). We previously characterized the catalytic subunit of the cryptococcal CK2 complex, Cka1, as a part of a systematic functional analysis of kinases in *C. neoformans* (29). *CKA1* deletion significantly reduces the growth of *C. neoformans* and affects normal cellular morphology, leading to a significant attenuation of infectivity and virulence (29). Recently, we also solved the crystal structure of Cka1 and found a dynamic architecture of the N-robe of Cka1 orthologs across species and deviation of the ATP binding pocket (30). Although *C. neoformans* Cka1 can bind to CX-4945, its IC_50_ is much higher than that of human CK2α, explaining why CX-4945 cannot inhibit the growth of *C. neoformans*. However, this finding suggests that the chemical derivatization of CX-4945 through structure-activity relationship (SAR) studies could allow the development of a cryptococcal CK2-specific inhibitor.

In this study, we comprehensively analyzed the molecular and pathobiological functions of the whole cryptococcal CK2 complex, consisting of a single catalytic subunit (Cka1) and two regulatory subunits (Ckb1 and Ckb2). Our study reveals that Cka1 and Ckb1/2 play major and minor roles, respectively, in diverse pathobiological functions of the CK2 complex and regulate many functionally diverse genes in *C. neoformans*. Surprisingly, we found that the CK2 complex is a master regulator of multiple signaling pathways, such as the Hog1 and Mpk1 MAPKs, calcium/calcineurin, and unfolded protein response (UPR) pathways, all of which are required for the pathogenicity of *C. neoformans*.

## RESULTS

### Identification of the CK2 complex and its pivotal role in the growth, cell cycle, and morphogenesis of *C. neoformans*

**Fig 1:**
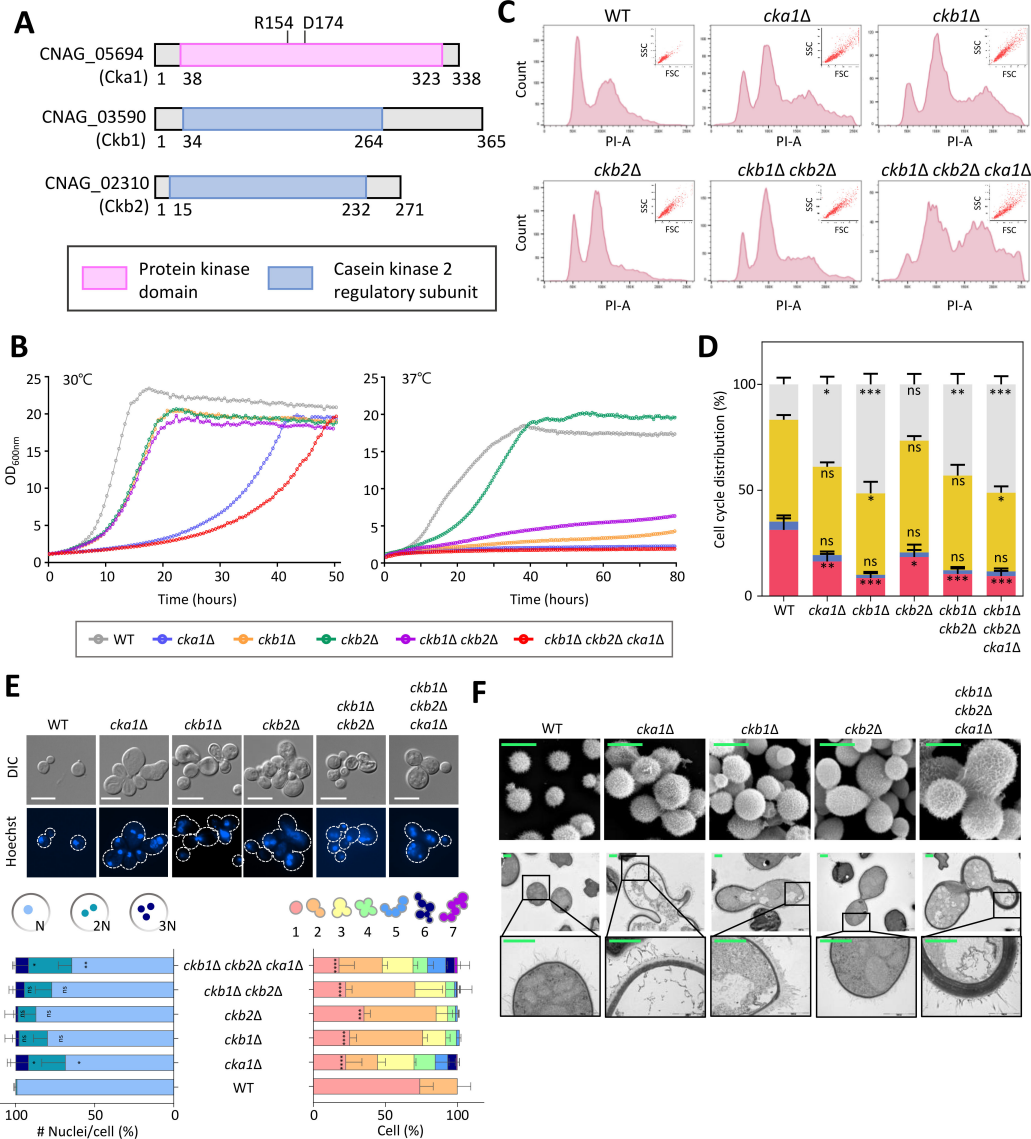
Roles of the CK2 complex in *C. neoformans* growth and morphology. (**A**) Domain analysis of CK2 complex subunits was conducted through searches on Pfam (http://pfam-legacy.xfam.org/) and UniProt (https://www.uniprot.org/). (**B**) Growth rates for wild-type (WT; H99S) and CK2 complex mutants [*cka1*Δ (YSB3052), *ckb1*Δ (YSB6680), *ckb2*Δ (YSB6727), *ckb1*Δ *ckb2*Δ (YSB6945), and *ckb1*Δ *ckb2*Δ *cka1*Δ (YSB7093)] were determined at 30°C and 37°C using a multi-channel bioreactor. (**C**) Cell cycle assessment of cells stained with propidium iodide (PI) using flow cytometry. The graph represents data from three biological replicates (see Fig. S2I). (**D**) Flow cytometry data segregated cell populations into G1, S, and G2/M phases, as detailed in Fig. S2I. Population proportions from three biological replicates were quantified. One-way ANOVA was used to assess statistical differences between wild-type and CK2 mutants. Statistical significance levels are indicated: ns (not significant), * (0.01 < *P* < .05), ** (0.001 < *P* < .01), *** (0.01 < *P* < .001). (**E**) Morphological evaluation of wild-type and CK2 mutants. Cells were fixed and stained with Hoechst (Scale bar = 10 µm). For nuclear count per cell, 50 cells were analyzed. The counts for single cells and interconnected cells spanned 50 cells each for wild-type and CK2 mutants (*N* = 50). The single cells of the wild-type and CK2 mutants were statistically compared. Data are depicted as mean ± standard errors of the means (SEM). One-way ANOVA determined statistical significance (ns, not significant; *, 0.01 < *P* < .05; **, 0.001 < *P* < .01; ***, 0.01 < *P* < .001; ****, *P* < 0.0001). (**F**) Electron microscopy image of wild-type and CK2 mutants. Cells underwent fixation with 2% of paraformaldehyde. The top panel showcases a scanning electron microscopy (SEM) image (scale bar: 5 µm), while the bottom panel offers a transmission electron microscopy (TEM) image (scale bar: 1 µm).

We initially analyzed the components of the fungal CK2 complex bioinformatically using the fungal genome database, FungiDB (http://fungidb.org/fungidb/). Fungal species typically possess a single CK2 catalytic subunit, with exceptions noted in *S. cerevisiae*, *Candida* spp., and *Mucor* spp. In *S. cerevisiae* and *C. albicans*, Cka1 and Cka2, the reported CK2 catalytic subunits, exhibit high sequence similarity (see Fig. S1A). In fungal species featuring only one CK2 catalytic subunit, Cka1 is evolutionarily conserved. Interestingly, *Mucor circinelloides* possesses four CK2 catalytic subunits. In contrast to humans, which have only one regulatory subunit, CK2β, fungal species generally have two distinct regulatory subunits, Ckb1 and Ckb2 (see Fig. S1B). Phylogenetically, human CK2β is closer to fungal Ckb1 than to Ckb2. Bioinformatic analysis revealed a single CK2 catalytic subunit, Cka1 (CNAG_05694), and two regulatory subunits, Ckb1 (CNAG_03590) and Ckb2 (CNAG_02310), in *C. neoformans* (see Fig. S1B). A protein domain analysis conducted using Pfam and Uniprot confirmed that Ckb1 and Ckb2 contain a CK2 regulatory subunit domain (Fig. 1A).

To analyze the function of the cryptococcal CK2 complex, we generated two independent deletion mutants for the *CKB1* and *CKB2* genes in the *MAT*α *C. neoformans* H99 strain background (see Fig. S2A and B). This was in addition to previously constructed *cka1*Δ mutants (29). We also generated the CK2 regulatory subunits double deletion mutants (*ckb1*Δ *ckb2*Δ), and a mutant with all CK2 subunits deleted (*ckb1*Δ *ckb2*Δ *cka1*Δ) (see Fig. S2C and D). To validate the mutant, we generated complemented strains for each mutant by integrating the wild-type allele into each gene’s native locus (see Fig. S2E through G).

As previously reported (29), the *cka1*Δ mutant exhibited severe growth defects at 30°C, and this growth defect was more pronounced at 37°C. The *ckb1*Δ and *ckb2*Δ mutants also displayed growth defects at 30°C and 37°C. Although the *ckb1*Δ mutant exhibited more pronounced growth defects than the *ckb2*Δ mutant at 37°C, both regulatory subunit mutants displayed less severe growth defects at 30°C and 37°C compared to those in the *cka1*Δ mutant (Fig. 1B). Each complemented strain demonstrated growth patterns comparable to the wild-type strain (see Fig. S3D). The *ckb1*Δ *ckb2*Δ double mutant exhibited a phenotype similar to the *ckb1*Δ mutants (Fig. 1B), suggesting that Ckb1 performs dominant roles among the regulatory subunits. The *ckb1*Δ *ckb2*Δ *cka1*Δ triple mutant showed even more severe growth defects than *cka1*Δ (Fig. 1B). These results suggest that the CK2 complex plays a pivotal role in controlling the growth of *C. neoformans* with Cka1 being more critical than Ckb1 and Ckb2.

Given the CK2 complex’s crucial function in the growth of *C. neoformans*, we hypothesized that CK2 may also be involved in the cell cycle control and morphogenesis of the fungus. To investigate this, we conducted fluorescence-activated cell sorting (FACS) analysis to quantify DNA content (side scatter, SSC) and cell size (forwarder scatter, FSC) in the wild-type and CK2 mutant strains. The wild-type strain exhibited regular G1, S, and G2 phases, while all CK2 mutants demonstrated abnormal cell cycle patterns (Fig. 1C). CK2 mutants had a pronounced decrease in the G1 phase and an increase in DNA content beyond 2N (Fig. 1C and D). In addition, both the DNA content and cell size for each CK2 mutant surpassed those of the wild-type strain (Fig. 1C). The morphological assessments and quantitative analyses corroborated that all CK2 mutants exhibited a significantly greater number of cells with multiple nuclei and interconnected morphologies than the wild-type strain (Fig. 1E). Among the mutants, the *cka1*Δ and *ckb1*Δ *ckb2*Δ *cka1*Δ mutants displayed the most evident defects (Fig. 1E), underscoring Cka1’s pivotal role in cell cycle and morphogenesis within the CK2 complex. Moreover, the more severe defects observed in the *ckb1*Δ mutant compared to the *ckb2*Δ mutant suggest that Ckb1 has a more substantial regulatory function than Ckb2.

Scanning and transmission electron microscopy provided further support for these observations. The CK2 mutants displayed abnormal cell morphologies, frequently presenting multiple interconnected cells, which could be attributed to cytokinesis defects (Fig. 1F). Generally, these mutants exhibited swollen, elongated, and abnormally large cell morphologies. Among them, the *cka1*Δ and *cka1*Δ *ckb1*Δ *ckb2*Δ mutants exhibited the most pronounced morphological abnormalities compared to the *ckb1*Δ and *ckb2*Δ mutants. Collectively, these findings indicate the pivotal role of the CK2 complex in controlling growth, cell cycle progression, and morphogenesis in *C. neoformans*. Within this complex, the catalytic subunit Cka1 plays a primary role, while the regulatory subunits Ckb1/2 have subsidiary functions.

### Physical interaction and nuclear localization of CK2 components in *C. neoformans*

**Fig 2:**
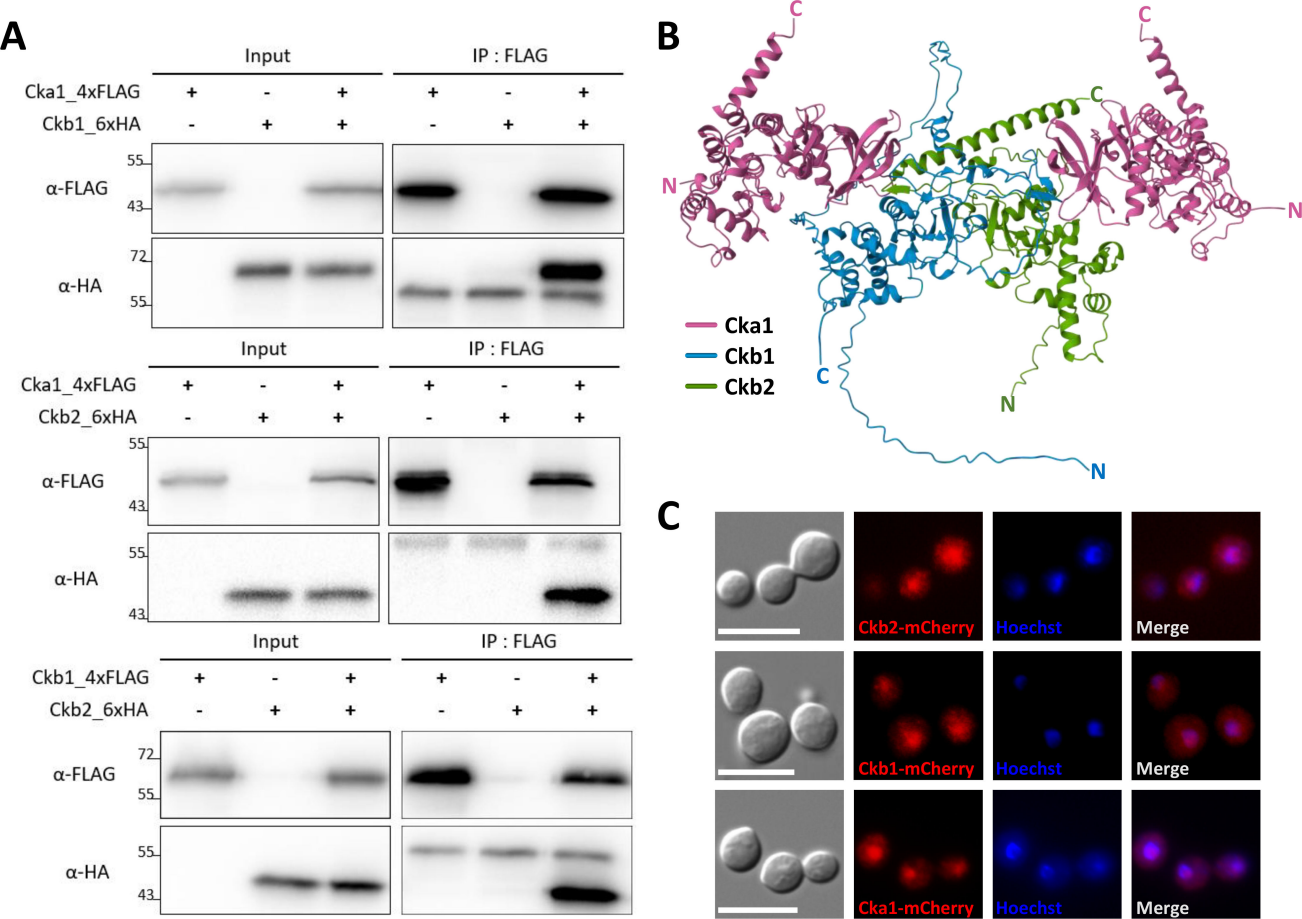
Cellular localization and *in vivo* interaction of CK2 subunits. (**A**) *In vivo* interaction among CK2 components. Whole cell lysates extracted from each strain—*cka1*Δ::*CKA1-4xFLAG-NEO* (YSB7784), *ckb1*Δ::*CKB1-4xFLAG-NEO* (YSB7812), *ckb1*Δ::*CKB1-6xHA-NEO* (YSB8251), *ckb2*Δ::*CKB2-6xHA-NEO* (YSB7846), *cka1*Δ::*CKA1-4xFLAG-NEO CKB1:6xHA-HYG* (YSB8081), *cka1*Δ::*CKA1-4xFLAG-NEO CKB2:6xHA-HYG* (YSB8179), and *ckb1*Δ::*CKB1-4xFLAG-NEO CKB2:6xHA-HYG* (YSB8082)—were subjected to immunoprecipitation using anti-FLAG antibody (IP; α-FLAG). The presence of 6x-HA-tagged proteins was subsequently verified by western blotting using an anti-HA antibody (IB; α-HA). (**B**) The predicted structure of the cryptococcal CK2 complex by AlphaFold 2 (ColabFold v1.5.2). Protein sequences were processed in the order of Cka1-Ckb1-Ckb2-Cka1. The color coding is as follows: Cka1 in pink, Ckb1 in blue, and Ckb2 in green. (**C**) Subcellular localization of CK2 components. Cells featuring mCherry-tagged CK2 components—*cka1*Δ::*CKA1-mCherry* (YSB7567), *ckb1*Δ::*CKB1-mCherry* (YSB6836), and *ckb2*Δ::*CKB2-mCherry* (YSB6821)—were fixed and stained with Hoechst dye to visualize the nucleus. Scale bar = 10 µm.

To ascertain the physical interactions between Cka1 and Ckb1/2, forming the CK2 complex, we assessed *in vivo* protein-protein interaction among the CK2 components. This was executed *via* co-immunoprecipitation (Co-IP) analysis using epitope-tagged strains of Cka1 and Ckb1/2 in *C. neoformans*. Specifically, we generated *C. neoformans* (H99) strains that expressed Cka1-4xFLAG, Ckb1-4xFLAG, Ckb1-6xHA, or Ckb2-6xHA and co-tagged strains in every feasible combination (see Fig. S3A through F). The epitope-tagged CK2 components appeared functional, as evident from the successful restoration of the normal growth patterns of their respective mutants under various stress inducers when complemented with each tagged allele (see Fig. S3G). Co-IP results indicated that Cka1 could bind both Ckb1 and Ckb2 regulatory subunits (Fig. 2A). Moreover, a mutual interaction between these regulatory subunits was detected (Fig. 2A). Consequently, our data support the notion that the cryptococcal CK2 complex consists of Ckb1 and Ckb2 forming a heterodimer and Cka1 binding to each of these regulatory subunits. Our Co-IP data suggest that the CK2 complex in *C. neoformans* may form a trimeric or tetrameric structure. Given the established tetrameric structure of human CK2, as determined by X-ray crystallography (3, 31), the CK2 complex in *C. neoformans* is also likely to adopt a tetrameric configuration. Supporting this, the AlphaFold 2-predicted structure of the cryptococcal CK2 complex is tetrameric (Fig. 2B). In previous research, the structure of *C. neoformans* Cka1 was elucidated and shown to bear similarity to human CK2α (30). By contrast, the structures of *C. neoformans* Ckb1 and Ckb2 diverged from that of human CK2β (see Fig. S3H). Ckb1 possesses a notably extended N- and C-terminal region compared to human CK2β. Though Ckb2 shares more structural similarities with human CK2β than Ckb1 does, it stands out with a prominently elongated α helix in its C-terminus. Supporting this, AlphaFold 2 predictions suggest that the overall tetrameric structure of the *C. neoformans* CK2 complex mirrors its human homolog but features distinct differences in the heterodimeric arrangement of the regulatory subunits.

Subsequently, we investigated the cellular localization of the CK2 complex in *C. neoformans*. For this purpose, the *cka1*∆, *ckb1*∆, and *ckb2*∆ mutants were complemented with the corresponding mCherry-tagged alleles—Cka1-mCherry, Ckb1-mCherry, and Ckb2-mCherry. Each mCherry allele was in-frame fused to the C-terminus of its respective wild-type allele (see Fig. S4A through C). All complemented strains replicated the growth rates of the wild-type strain, demonstrating the functionality of the mCherry-tagged proteins (see Fig. S4D). Significantly, we discerned a pronounced nuclear enrichment of all mCherry-tagged CK2 components (Fig. 2C), a finding that harmonizes with the CK2 complex’s pivotal role in the cell cycle regulation of *C. neoformans*.

### Multifaceted roles of the CK2 complex in the pathobiology of *C. neoformans*

**Fig 3:**
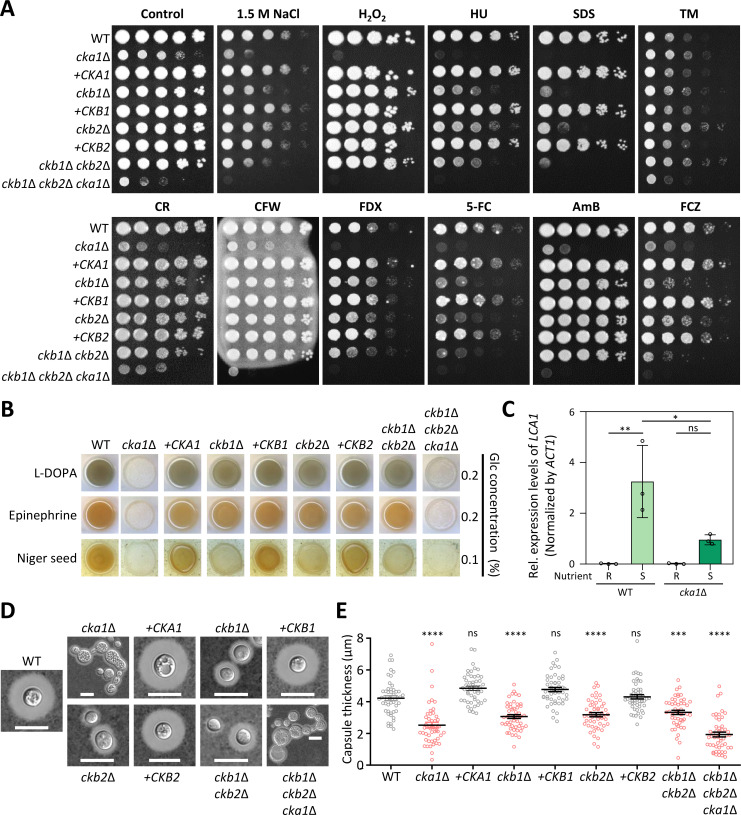
Multifaceted roles of CK2 in stress adaptation and virulence factor production in *C. neoformans*. (**A**) Stress susceptibility assay. Susceptibility of various strains, including wild-type (H99S), *cka1*Δ (YSB3052), *cka1*Δ::*CKA1* (YSB6746), *ckb1*Δ (YSB6680), *ckb1*Δ::*CKB1* (YSB6840), *ckb2*Δ (YSB6727), *ckb2*Δ::*CKB2* (YSB6826), *ckb1*Δ *ckb2*Δ (YSB6945), and *ckb1*Δ *ckb2*Δ *cka1*Δ (YSB7093), to antifungal agents and stress inducers were assessed. Strains were cultured overnight in YPD broth at 30°C, serially 10-fold diluted, and spotted onto YPD plates with stress inducers such as 1 M NaCl, 2 mM hydrogen peroxide (H_2_O_2_), 120 mM hydroxyurea (HU), 0.02% sodium dodecyl sulfate (SDS), 0.4 µg/mL tunicamycin (TM), 0.8% Congo red (CR), 5 mg/mL calcofluor white (CFW), 5 µg/mL fludioxonil (FDX), 600 µg/mL flucytosine (5FC), 0.8 µg/mL amphotericin B (AmB), and 18 µg/mL fluconazole (FCZ). Growth was observed after a 5-day incubation at 30°C. (**B**) Melaninization assay. Melanin production was examined by spotting overnight cultured strains onto Niger seed, L-DOPA, and epinephrine agar media containing 0.1% or 0.2% glucose. (**C**) Gene expression analysis. Quantitative reverse transcription-PCR (qRT-PCR) for measuring *LAC1* induction was conducted utilizing total RNA from wild-type and CK2 mutant strains grown under nutrient-rich (R; YPD) or nutrient-limited (S; YNB without glucose) conditions. Data are based on three independent biological experiments, each with three technical replicates. The error bars represent SEM. Statistical significance in gene expression levels was evaluated using one-way ANOVA (ns, not significant; *, 0.01 < *P* < .05; **, 0.001 < *P* < .01). (**D and E**) Capsule visualization and quantification. Following induction in Littman’s medium (LIT) at 37°C over 2 days, capsule thickness was both visualized (**D**) and quantified (**E**). Images were captured with a scale bar set at 10 µm, and measurements were taken from 50 cells per strain (*n* = 50). Statistical significance was determined using one-way ANOVA with Bonferroni’s multiple comparison test. Data are displayed as mean ± SEM, with significance levels indicated: ns, not significant; ***, 0.01 < *P* < .001; ****, *P* < 0.0001.

We delved into the diverse pathobiological functions of the CK2 complex in *C. neoformans*. Initially, we examined its role in stress response and adaptation in the pathogen. We evaluated the growth defects of CK2 mutants under unstressed control conditions and then compared their growth inhibition to that of the wild-type strain in both the presence and absence of various stressors *via* spot assays. The *cka1*Δ, *ckb1*Δ, and *ckb2*Δ mutants were more susceptible to multiple external stresses, such as osmotic, oxidative, and genotoxic stresses, as well as destabilizing agents for cell walls/membranes and various antifungal treatments (Fig. 3A). Typically, the *cka1*Δ mutant was more susceptible to these stresses than *ckb1*Δ, *ckb2*Δ, or *ckb1*Δ *ckb2*Δ mutants, with the notable exception of the ER stress agent tunicamycin. The *cka1*Δ *ckb1*Δ *ckb2*Δ triple mutant displayed an even more extreme stress susceptibility than *cka1*Δ. Complementation with each wild-type allele successfully restored stress resistance levels in the CK2 mutants. Interestingly, all mutants displayed increased resistance to tunicamycin, except the *cka1*Δ *ckb1*Δ *ckb2*Δ triple mutant exhibiting increased susceptibility (Fig. 3A). Upon extended incubation of 6 days, the heightened stress susceptibility of both the *cka1*Δ and *cka1*Δ *ckb1*Δ *ckb2*Δ mutants become more pronounced, as depicted in Fig. S5A. The observations from these qualitative spot assays were substantiated by directly examining the CK2 complex’s influence on specific stress-responsive signaling pathways, such as Mpk1 and Hog1 MAPK and calcineurin pathways, which we detail in Fig. 7. Overall, Cka1 appears to have a dominant role in stress adaptation, with Ckb1/2 playing a supplementary role in *C. neoformans*.

We then assessed the ability of CK2 mutants to produce the antioxidant melanin pigment and antiphagocytic polysaccharide capsule, both crucial virulence factors in *C. neoformans*. All CK2 mutants displayed reduced melanin production, with *cka1*Δ and *ckb1*Δ *ckb2*Δ *cka1*Δ mutants presenting the most pronounced decrease (Fig. 3B). In *C. neoformans*, melanin production is typically triggered by carbon deprivation, leading to induction of the laccase gene (*LAC1*) expression (32, 33). Our data revealed that during glucose deprivation, the induction of *LAC1* in the *cka1*Δ mutant was significantly lower than in the wild-type strain (Fig. 3C). This points to CK2 as a likely positive regulator of *LAC1*.

Similarly, CK2 appeared instrumental in capsule production. Its absence resulted in diminished capsule output in *C. neoformans* (Fig. 3D and E). Specifically, the deletion of *CKA1* notably reduced capsule production under capsule-inducing conditions (Fig. 3D). This implies a positive role for Cka1 in capsule synthesis. Quantitative analyses further reinforced this notion, displaying significantly reduced capsule production in the *cka1*Δ and *ckb1*Δ *ckb2*Δ *cka1*Δ mutants (Fig. 3E). In summary, our findings underscore the pivotal, multifaceted roles of the CK2 complex in modulating key pathobiological features of *C. neoformans*.

### Essential roles of the CK2 complex in the virulence of *C. neoformans*

Given the central role of the CK2 complex in diverse *in vitro* pathobiological features of *C. neoformans*, we turned our attention to the virulence potential of CK2 components within *C. neoformans*, employing insect and murine host models of systemic cryptococcosis. Using the insect model using *Galleria mellonella* (34), we previously showed that the *cka1*∆ mutants exhibit reduced virulence (29). Similarly, we found that *ckb1*∆ and *ckb2*∆ mutants showed decreased virulence, but their complemented strains were as virulent as the wild-type (see Fig. S5B). The *ckb1*∆ *ckb2*∆ double mutant also had reduced virulence. However, the *ckb1*∆ *ckb2*∆ *cka1*∆ triple mutant showed even more diminished virulence than the *ckb1*∆ *ckb2*∆ double mutant, emphasizing the significant role of Cka1 in the virulence of *C. neoformans* (see Fig. S5).

**Fig 4:**
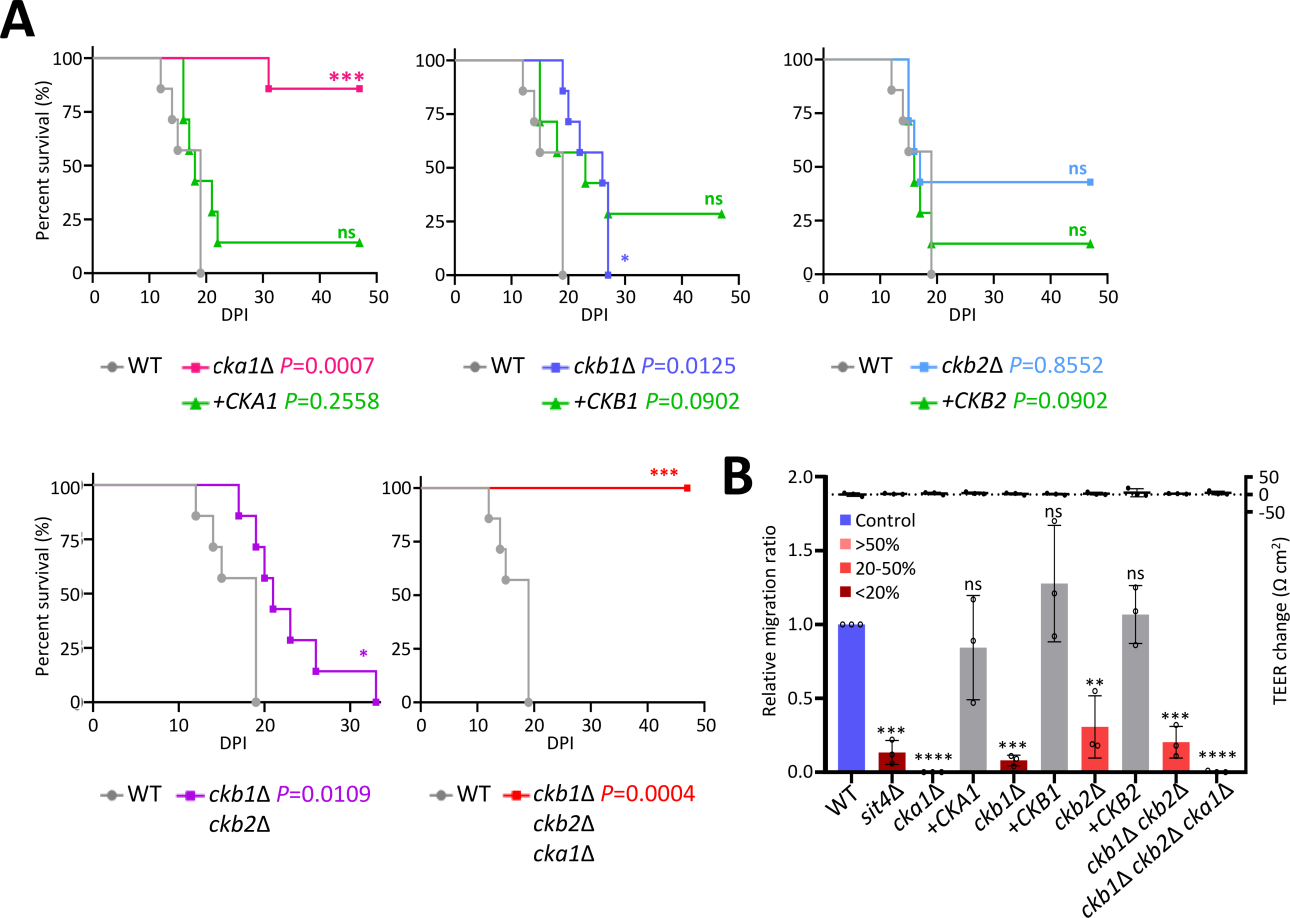
The crucial role of CK2 in *C. neoformans* pathogenicity. (**A**) Virulence assay in mice. BALB/c mice (*n* = 7 per group) were intranasally infected with *C. neoformans* strains: wild-type (**H99**), *cka1*Δ (YSB3052), *ckb1*Δ (YSB6680), *ckb2*Δ (YSB6727), *ckb1*Δ *ckb2*Δ (YSB6945), and *ckb1*Δ *ckb2*Δ *cka1*Δ (YSB7093). Mice were monitored daily for signs of morbidity. Survival curves were statistically analyzed using the log-rank (Mantel-Cox) test. (**B**) Blood-brain barrier (BBB) crossing assay. The ability of wild-type and CK2 mutants to cross the BBB was tested in an *in vitro* BBB transwell model. The *sit4*Δ mutant (YSB4095) was used as a control strain showing defective BBB crossing ability. Statistical significance was calculated using one-way ANOVA analysis with Dunnett’s multiple comparison test. (****, *P* < 0.0001; ***, 0.0001 < *P* < .0002; **, 0.0021 < *P* < .0332; ns, not significant)

In the systemic cryptococcosis murine model, the *cka1*∆ mutant presented a significant virulence reduction compared to wild-type and complemented strains (Fig. 4A). The *ckb1*∆ mutant showed slightly reduced virulence compared to the wild-type (*P* = 0.0125), although the restoration of virulence was not fully achieved through the complementation of *CKB1* gene (*P* = 0.09). The *ckb2*∆ mutants did not exhibit notable virulence diminution. In addition, the virulence profile of the *ckb1*∆ *ckb2*∆ double mutant was similar to that of the *ckb1*∆ mutant being less virulent than the wild-type (Fig. 4A), implying that Ckb1 may play a marginal role in virulence. As expected, the *ckb1*∆ *ckb2*∆ *cka1*∆ triple mutant displayed a complete virulence abrogation (Fig. 4A). Collectively, these data imply distinct major and minor virulence roles of Cka1 and Ckb1 in *C. neoformans*, respectively.

Given the fact that *C. neoformans* causes fatal brain infections in mammalian hosts, we probed the ability of the CK2 mutants to breach the blood-brain barrier (BBB). This was undertaken using a previously established *in vitro* BBB transwell model (35). Both *cka1*Δ and *ckb1*Δ *ckb2*Δ *cka1*Δ triple mutants displayed a complete loss of BBB crossing ability. By contrast, *ckb1*Δ and *ckb2*Δ mutants exhibited a reduced transgression capability, approximating 20% of the wild-type’s capability (Fig. 4B). Moreover, transendothelial electrical resistance (TEER) values remained invariant pre- and post-inoculation with the wild-type or CK2 mutants (Fig. 4B), suggesting that the CK2 complex’s functionality does not impinge on the BBB’s tight junction integrity. These findings were well congruent with the systemic mouse infection data. In summation, these findings echo the systemic murine infection data and underscore the CK2 complex’s pivotal role in determining the pathogenicity of *C. neoformans*.

### Transcriptional networks governed by the cryptococcal CK2 complex

The nuclear-localization and wide-ranging roles of the CK2 complex in both *in vitro* and *in vivo* pathobiological functions of *C. neoformans* hint at its potential regulation of a myriad of transcriptional networks. To delineate this, we conducted an RNAseq-based transcriptome profiling analysis of *cka1*∆ and *ckb1*∆ *ckb2*∆ *cka1*∆ strains, comparing them to the wild-type. The coherence of each biological replicate affirms the reliability of our transcriptome data (see Fig. S6A through C).

**Fig 5:**
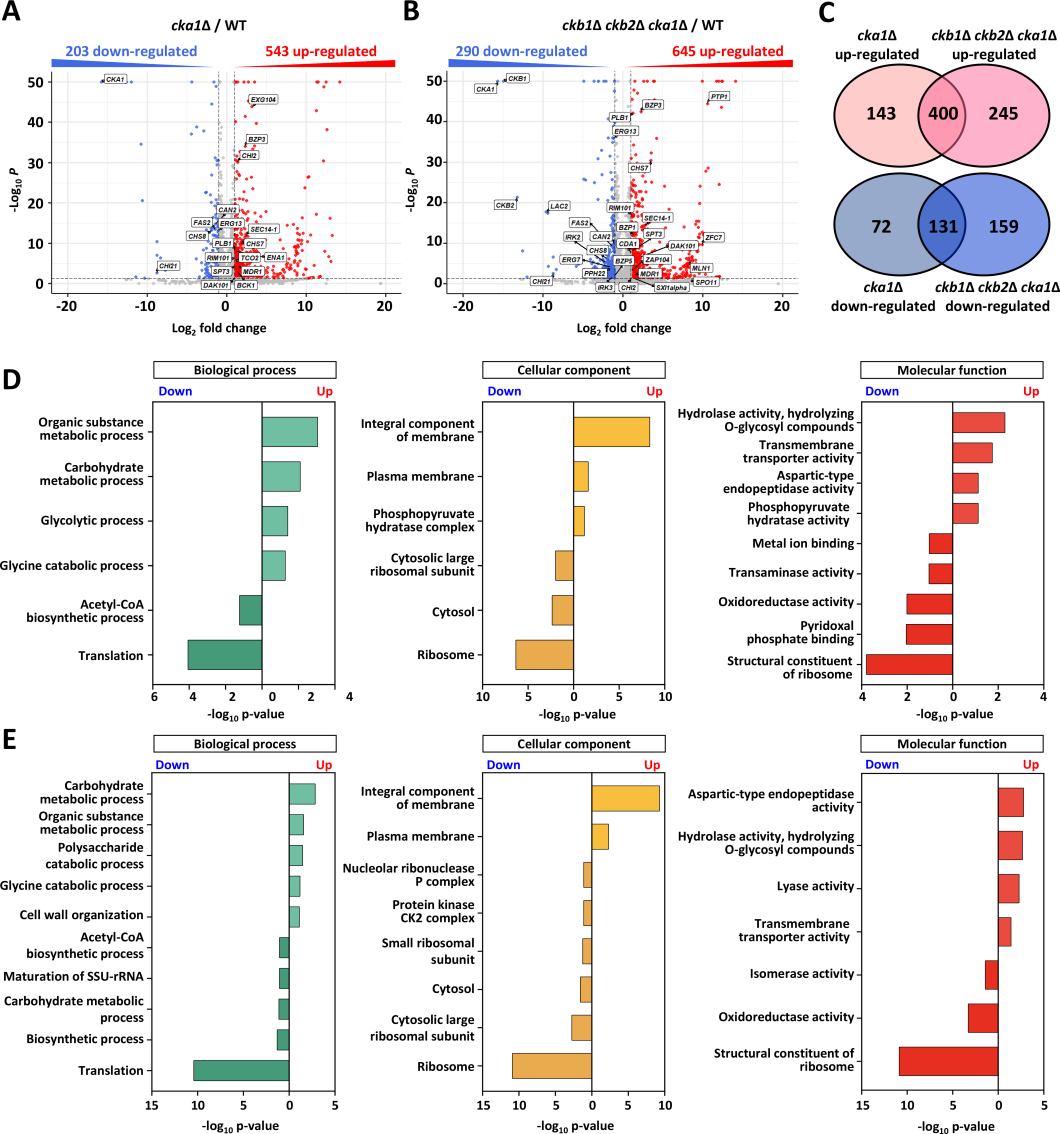
Transcriptional profiles modulated by the CK2 complex in *C. neoformans*. (**A and B**) RNA sequencing-based transcriptome analysis. Volcano plots illustrate the variation in gene expression levels in *cka1*Δ (YSB3052) (**A**) or *ckb1*Δ *ckb2*Δ *cka1*Δ (YSB7093) (**B**) mutants in comparison to the wild-type. Genes exhibiting significant upregulation are marked in red, while downregulated genes are highlighted in blue. (**C**) Venn diagrams depict the number of genes with altered expression—either upregulated or downregulated—in the *cka1*Δ and/or *ckb1*Δ *ckb2*Δ *cka1*Δ mutants compared to the wild-type. (**D and E**) Functional categorization of differential expression. The DAVID analysis tool was utilized to classify differentially expressed genes based on functional categories for both *cka1*Δ (**D**) and *ckb1*Δ *ckb2*Δ *cka1*Δ (**E**) mutants.

Remarkably, *CKA1* deletion led to significant expression shifts (>2 fold cutoff; *P* < 0.05) in 746 genes, with 203 being downregulated and 543 upregulated, which equates to 10.2% of *C. neoformans* genes (Fig. 5A). Furthermore, the collective deletion of all CK2 subunits impacted more genes (935 genes in total, with 290 downregulated and 645 upregulated) than the single deletion of *CKA1* (Fig. 5B). Though significant overlaps existed in upregulated (400) and downregulated (131) genes existed between *cka1*∆ and *ckb1*∆ *ckb2*∆ *cka1*∆ strains, distinct transcriptomic footprints were evident (Fig. 5C), alluding to potential transcriptional contributions by Ckb1 and Ckb2.

Gene ontology (GO) analysis helped elucidate the biological processes, cellular components, and molecular functions of genes regulated by *CKA1* (Fig. 5D) and the CK2 complex (Fig. 5E). Echoing the observed growth anomalies in CK2 mutants, genes influenced by the CK2 complex were predominantly linked to metabolic processes of carbohydrates, amino acids, and organic substances. Concurrently, an upregulation of transmembrane transporters by CK2 deletion hinted at an augmented quest for more external sources (Fig. 5D and E; Data Set S2). Aligning with the CK2 complex’s pivotal role in stress adaptation, our data revealed its regulatory role over genes involved in various stress reactions. This includes genes like *TCO2*, associated with external stress signals *via* the Hog1 MAPK pathway (36), and *ENA1*, a cation transporter modulated by Hog1 during osmotic stress (37). In addition, *BCK1* expression, crucial to the cell wall integrity Mpk1 MAPK pathway, was enhanced post*-CKA1* deletion (Fig. 5A; Data Set S2). Given CK2’s nuclear localization, it seems to orchestrate the expression of pivotal transcription factors, such as *MLN1*, *BZP3*, *SXI1alpha*, *RIM101*, and *ZAP104*, thereby modulating diverse pathobiological signaling pathways (Fig. 5B; Data Set S2). In summary, our transcriptomic data underscore the CK2 complex’s profound influence over a plethora of metabolic and stress-responsive transcriptional networks in *C. neoformans*.

### Phosphoproteome profiles governed by the cryptococcal CK2 complex

**Fig 6:**
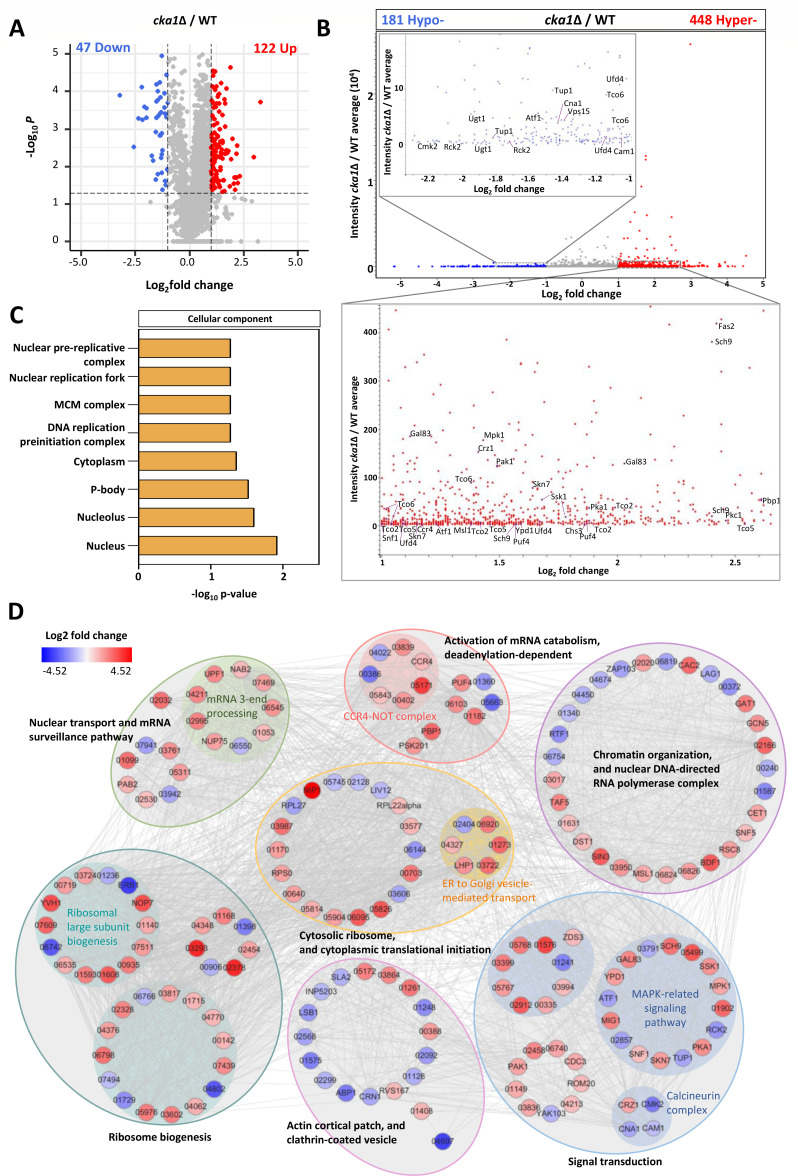
Global proteomic and phosphoproteomic analyses of Cka1. (**A**) Differential protein expression analysis. A volcano plot visualizes proteins based on their differentially expressed proteins (DEPs) and corresponding *P* values. Points on the plot represent individual proteins, with the x-axis indicating fold changes in expression and the y-axis representing the statistical significance as −log10 of the associated *P* values. (**B**) Phosphopeptide intensity analysis. This volcano plot illustrates changes in phosphopeptide intensity in *cka1*Δ relative to the wild-type. The overarching trends are portrayed in the top panel. Specific regions enriched for either increased (hyper-) or decreased (hypo-) phosphorylation are magnified in the panels beneath and within the main graph, respectively. (**C**) Phosphoproteomic functional categories. The DAVID tool was employed to categorize and analyze the phosphoproteomics data set of *cka1*Δ based on functional classifications. (**D**) Phosphorylation-driven protein interaction networks. Proteins exhibiting altered phosphorylation states in *cka1*Δ were evaluated using STRING (http://string-db.org) to predict potential protein-protein interactions. The resulting network was visualized using Cytoscape v3.10.0.

Recognizing Cka1 in the CK2 complex as a kinase, we sought to identify potential Cka1 phosphorylation targets using proteomics and phosphoproteomics analyses. Employing higher-resolution liquid chromatography-tandem mass spectrometry (LC-MS/MS) coupled with quantitative proteomics, we profiled the *cka1*∆ mutant (see Fig. S6E). Our proteomic analysis identified a total of 3,502 proteins, quantifying 2,798. Of these, 318 proteins were differentially expressed: 259 upregulated and 59 downregulated (see Fig. S6F). With a Pearson correlation coefficient exceeding 0.769 across three biological replicates, the consistency of our results was evident (see Fig. S6G). Specifically, 122 proteins exhibited upregulation with a log_2_ fold-change greater than 1 (*P* < 0.05), and 47 were downregulated with a log_2_ fold-change less than −1 (*P* < 0.05) (Fig. 6A; Data Set S3). We subsequently assessed phosphorylation level shifts between wild-type and *cka1*∆ strains, normalized against quantified protein levels in the proteomics data (Fig. 6B). This normalized phosphoproteomics analysis revealed 448 hyperphosphorylated and 181 hypophosphorylation sites (>2-fold cutoff; *P* < 0.05) upon *CKA1* deletion.

To unveil phosphoproteome linkages between CK2 and other signaling pathways, we turned to STRING (search tool for the retrieval of interacting genes/proteins) analysis (Fig. 6C and D). Notably, the CK2 complex appeared to modulate numerous metabolic and signaling pathways *via* phosphorylation. Given the CK2 complex’s nuclear enrichment and its transcriptome-wide influence, we observed the CK2-dependent post-translational regulation of proteins implicated in mRNA processing, surveillance, and decay. Examples encompass the CCR4-NOT4 complex, a player in mRNA quality control and chromatin remodeling (38). In addition, CK2’s influence extends to translation, modulating ribosome biogenesis and cytoplasmic translational initiation (Fig. 6D).

Most strikingly, the CK2 complex seems to directly orchestrate myriad pathobiological signaling pathways vital for *C. neoformans* virulence (Fig. 6D). Upon *CKA1* deletion, phosphorylation shifts were observed in the calcineurin pathway’s key components, such as Cam1, Cna1, Cmk2, and Crz1, and the Hog1 MAPK pathway components, like Tco1, Ypd1, Skn7, Ssk1, and Atf1 (Fig. 6D). Moreover, phosphorylation changes in Pka1 and Msl1, pivotal in the cAMP/PKA signaling pathway, were detected (Fig. 6D). Reflecting CK2’s influence on cell growth and morphogenesis, altered phosphorylation was detected in the cell wall integrity Mpk1 MAPK pathway components, such as Pkc1 and Mpk1. In summary, the CK2 complex appears to post-translationally steer a plethora of pathobiological signaling pathways central to *C. neoformans* pathogenicity.

### CK2 disruption leads to dysregulation of key cryptococcal pathobiological signaling pathways

Our combined transcriptomic and phosphoproteomic findings propose that the CK2 complex may act as an overarching regulator for cryptococcal signaling pathways intrinsic to *C. neoformans* virulence. To validate this hypothesis experimentally, we studied the role of the CK2 complex in orchestrating the Mpk1 and Hog1 MAPK, unfolded protein response (UPR), and calcineurin/Crz1 signaling pathways—each acknowledged as crucial for *C. neoformans* virulence (39, 40).

**Fig 7:**
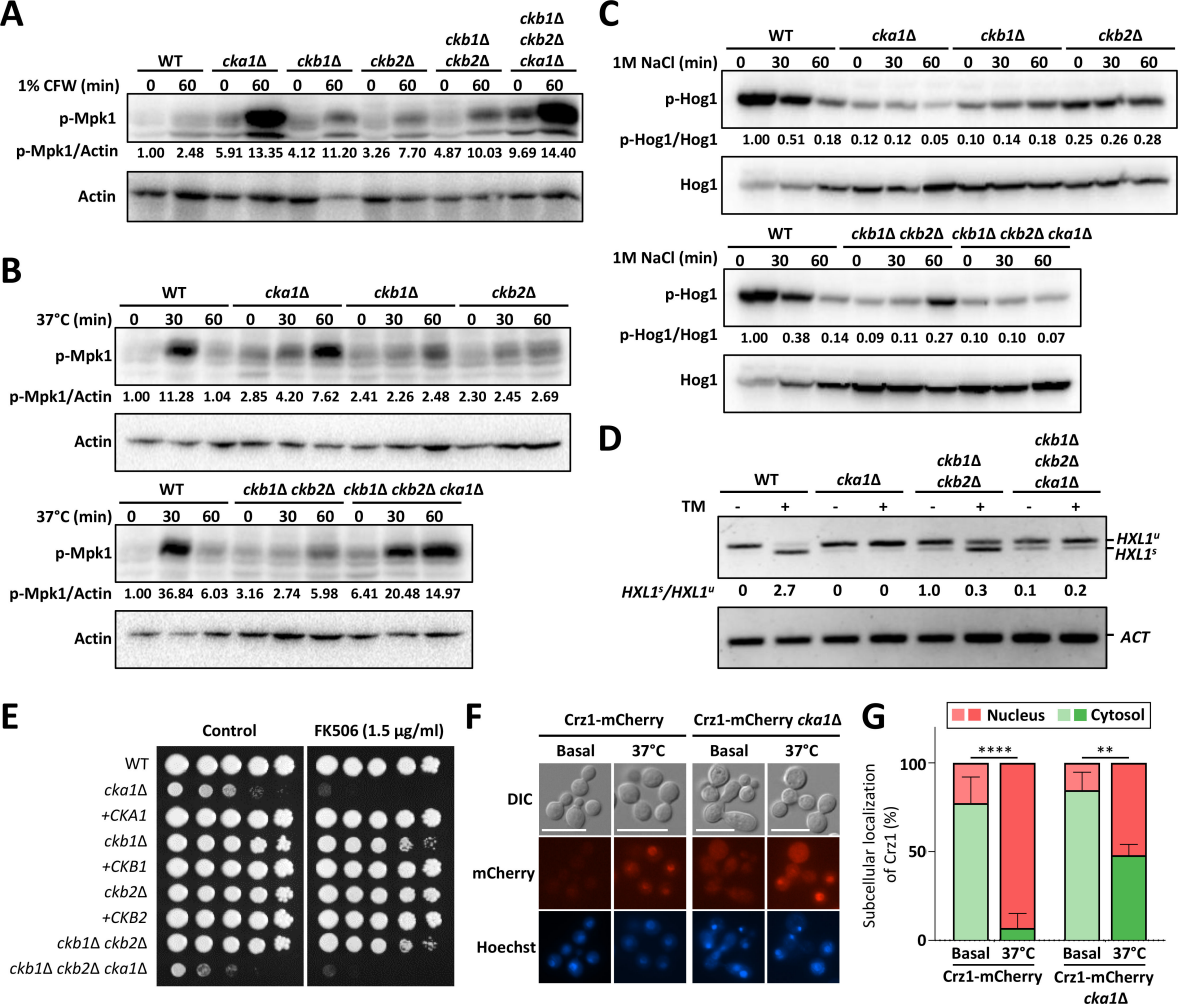
Role of CK2 in mediating pathobiological signaling in *C. neoformans*. (**A–C**) Wild-type (**H99**), *cka1*Δ (YSB3052), *ckb1*Δ (YSB6680), *ckb2*Δ (YSB6727), *ckb1*Δ *ckb2*Δ (YSB6945), and *ckb1*Δ *ckb2*Δ *cka1*Δ (YSB7093) strains were cultivated until the mid-logarithmic phase, following which they were exposed to specific stressors for defined durations. Subsequently, protein extracts were prepared for western blot analysis. (**A and B**) Mpk1 phosphorylation was induced by 1% calcofluor white (CFW) (**A**) and 37°C (**B**) stress. Western blots were conducted using the anti-phospho-p44/p42 antibody, with β-actin serving as a loading control. (**C**) Cells exposed to 1 M NaCl showed variations in Hog1 phosphorylation levels, as visualized using the anti-P-p38 antibody. The anti-Hog1 polyclonal antibody acted as a loading reference. (**D**) RT-PCR was employed to monitor the splicing behavior of *HXL1* in the presence of ER stress. After exposing the wild-type and CK2 mutant strains to 5 µg/mL of TM for 1 h, cDNA was synthesized from the extracted total RNA. (**E**) FK506 sensitivity of CK2 mutants. Following overnight growth, wild-type and CK2 mutant strains were serially diluted and spotted on YPD plates, either containing FK506 or not. Post-incubation at 30°C, growth was observed over 4 days. (**F and G**) Cellular localization of Crz1-mCherry in the *CRZ1-mCherry* (JL334) and *CRZ1-mCherry cka1*Δ (YSB11149) strains were studied after a temperature shift from 23°C to 37°C over 1 h using DIC microscopy (BX51, Olympus). (**G**) The bar chart displays the proportion of cells exhibiting Crz1-mCherry localization either in the nucleus or cytoplasm. A sample of 100 cells were counted (*n* = 100). Statistical significance was determined using one-way ANOVA analysis, accompanied by Tukey’s multiple comparison test. Data are displayed as mean ± SEM (**, 0.001 < *P* < .01; ****, *P* < 0.0001).

First, we delved into CK2’s impact on the cell wall integrity Mpk1 MAPK pathway. Mpk1 exhibited a twofold to threefold phosphorylation enhancement in the wild-type strain post calcofluor white (CFW) treatment—a cell wall-disrupting agent (Fig. 7A). Intriguingly, basal Mpk1 phosphorylation elevated considerably across all CK2 mutants (Fig. 7A), suggesting potential cell wall integrity compromise in CK2’s absence. In the *cka1*∆ and *ckb1*∆ *ckb2*∆ *cka1*∆ mutants, CFW induced a pronounced surge in Mpk1 phosphorylation levels—6–7 times that of the wild-type (Fig. 7A). Notably, a temperature shift to 37°C from 30°C led to heightened Mpk1 phosphorylation (over 10-fold) in the wild-type strain, but this elevation was less pronounced or delayed in the CK2 mutants (Fig. 7B). These results indicate that the CK2 complex may play unique modulatory role in the Mpk1 MAPK pathway upon distinct cell wall stresses.

Second, we explored CK2’s influence on the stress-activated Hog1 MAPK pathway. In the *C. neoformans* H99 strain, Hog1 is predominantly phosphorylated under basal conditions and becomes dephosphorylated upon osmotic stress (39) (Fig. 7C). Strikingly, basal Hog1 phosphorylation levels were significantly reduced, especially evident in the *cka1*∆ and *ckb1*∆ *ckb2*∆ *cka1*∆ mutants (Fig. 7C). Upon 1 M NaCl exposure, Hog1 phosphorylation remained relatively unchanged across CK2 mutants (Fig. 7C). Such observations intimate CK2’s critical role in maintaining the integrity of the Hog1 MAPK signaling in *C. neoformans*.

Third, we assessed CK2’s regulatory capacity within the UPR pathway. The UPR pathway in *C. neoformans* encompasses the Ire1 kinase/endoribonuclease and Hxl1 transcription factor (41). When exposed to ER stress agents like tunicamycin (TM), Ire1 mediates unconventional splicing in the native *HXL1* transcript (*HXL1^u^*) to generate an activated, spliced *HXL1* transcript (*HXL1^s^*). This activated Hxl1 subsequently triggers various downstream components, like the Kar2 molecular chaperone, to mitigate the ER stress (42). Our analyses revealed a pronounced accumulation of the unspliced *HXL1* in the *cka1*∆ and *ckb1*∆ *ckb2*∆ *cka1*∆ mutants independent of ER stress (Fig. 7D). In the *ckb1*∆ *ckb2*∆ mutant, TM treatment induced a noticeable delay in converting *HXL1* to its spliced form.

Lastly, we examined the influence of CK2 disruption on the calcineurin/Crz1 pathway, which is pivotal for cell wall integrity and thermotolerance in *C. neoformans*. In response to an external cue, intracellular Ca^2+^ binds to the calmodulin protein (Cam1), which then activates the calcineurin phosphatase complex (Cna1 and Cnb1). This activated calcineurin, in turn, dephosphorylates the transcription factor, Crz1, facilitating its nuclear translocation to regulate downstream genes (43). Our phosphoproteomics analysis revealed hyperphosphorylation of Cam1, Cna1, Cnb1, and Crz1 in the *cka1*∆ mutant (Fig. 6B and D). Corroborating these findings, we observed increased susceptibility to the calcineurin inhibitor FK506 in *cka1*∆, *ckb1*∆, and *ckb2*∆ mutants, suggesting that the disruption of CK2 interferes with calcineurin pathway regulation (Fig. 7E). Intriguingly, the residue S103 of Crz1, which becomes phosphorylated upon *CKA1* deletion, has been previously reported to be critical for Crz1 function (43). To further explore this, we deleted *CKA1* in a strain expressing Crz1-mCherry (44) and assessed Crz1’s nuclear translocation efficiency (see Fig. S7). The Crz1-mCherry protein was evenly distributed within the cytoplasm of cells grown under basal conditions (23°C). When subject to 37°C, a temperature that triggers calcineurin activation, Crz1 translocated to the nucleus. Yet, in the *cka1*Δ mutant cells, the translocation of Crz1-mCherry into the nucleus was less pronounced in response to the temperature increase (Fig. 7F and G).

In summary, our data strongly suggest that the CK2 complex serves as a central regulator of crucial pathobiological signaling pathways essential for the virulence of *C. neoformans*.

## DISCUSSION

**Fig 8:**
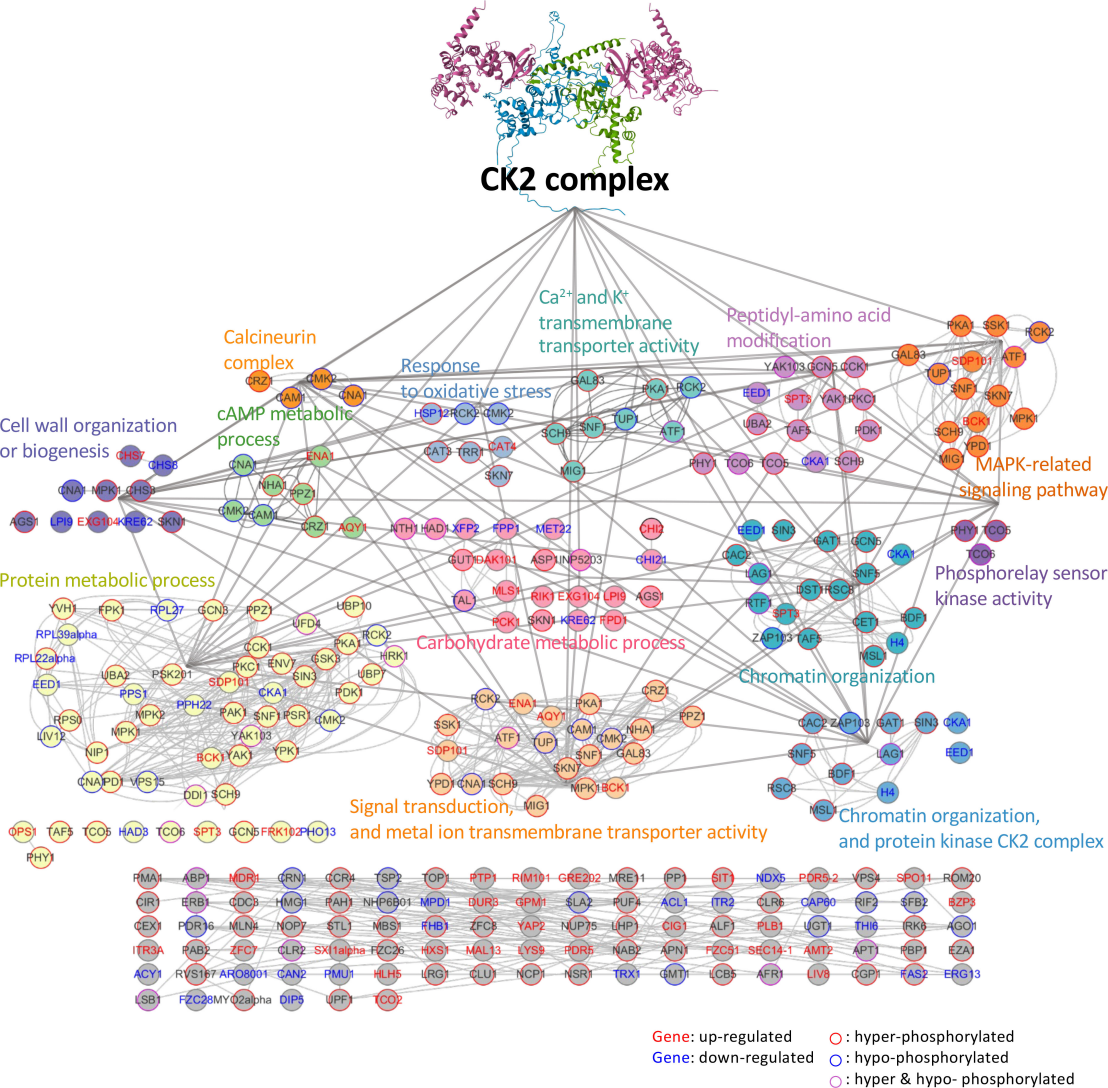
Proposed CK2-dependent signaling and metabolic pathways in *C. neoformans*. Annotated genes from the altered transcriptome and phosphoproteome of *cka1*Δ were analyzed using STRING (http://string-db.org) to predict possible protein-protein interactions. In the transcriptome analysis, genes exhibiting significant upregulation are marked in red, while downregulated genes are highlighted in blue. In the phosphoproteomics analysis, genes that are hyperphosphorylated are outlined in red, and those that are hypophosphorylated have a blue outline. Genes that are both hypo- and hyperphosphorylated in the same protein are indicated in purple outline. Genes associated with the same signaling pathway are marked in the same color. These predictive networks were displayed using Cytoscape v3.10.0.

This study elucidates the critical role and regulatory mechanisms of the CK2 complex in the globally significant fungal meningitis pathogen, *C. neoformans*. Disruption of the CK2 complex led to pronounced growth impairments, including delays in cell cycle progression and cytokinesis, reduced stress tolerance, and compromised antifungal drug resistance. These disruptions substantially affected the virulence of this basidiomycetous fungus. Through comprehensive transcriptome and phosphoproteomics analyses, supported by molecular and genetic investigations, we discovered that the CK2 complex orchestrates several key signaling cascades known to regulate the virulence of *C. neoformans*, including Hog1, Mpk1 MAPKs, cAMP/PKA, and calcineurin/Crz1 signaling pathways (Fig. 8). Our findings not only deepen our understanding of the multifaceted roles of the fungal CK2 complex but also offer promising avenues for the development of novel anticryptococcal agents targeting this complex.

The structural composition of the CK2 complex varies widely, even among fungi, despite its evolutionary conservation across eukaryotes. In mammals, including humans, the CK2 complex is typically composed of multiple catalytic subunits along with a single regulatory subunit. In ascomycetous yeasts such as *S. cerevisiae* and various *Candida* species, the complex contains two catalytic and two regulatory subunits. However, in other ascomycetous fungi like *Schizosaccharomyces pombe* and *Aspergillus* species, the CK2 complex consists of a single catalytic subunit and two regulatory subunits. A similar structural arrangement is also prevalent in most basidiomycetous fungi, including *C. neoformans* and *Ustilago maydis*. Therefore, the fungal CK2 complex is principally formed by one catalytic subunit and two regulatory subunits. A notable exception to these configurations is found in Mucorales. Our bioinformatics analyses suggest that *Mucor* species possess four catalytic subunits and five regulatory subunits. The unique composition in *Mucor* raises intriguing questions about how these multiple catalytic and regulatory subunits interact and whether distinct or redundant roles are played by different CK2 complexes within the same fungal species.

The essentiality of the fungal CK2 complex appears to differ among various fungi. Our study emphasizes that while CK2 plays crucial roles in a variety of pathobiological processes in *C. neoformans*, it is not vital for the organism’s survival. This is evident from our successful triple deletion of all CK2 subunits. Conversely, in *S. cerevisiae* and *C. albicans*, the CK2 complex is essential, as two catalytic CK2 subunits have a synthetic lethal relationship (45, 46). Even in *S. pombe*, which has only one catalytic subunit, Cka1 is essential for survival (47). In filamentous fungi, Cka1 is essential for *Aspergillus nidulans*, but not for *Neurospora crassa* (48). Consequently, the essential nature of the fungal CK2 complex continues to be a subject of investigation across different fungal pathogens.

In our study, we established that Cka1 engages in a physical interaction with both Ckb1 and Ckb2. Furthermore, interactions observed between the two regulatory subunits hint at a typical heterotetramer structure of the cryptococcal CK2 complex. However, the specifics of how these catalytic and regulatory subunits interplay and modulate each other remain to be delineated. In the human system, the sole regulatory subunit, CK2β, features an autophosphorylation site in its N-terminus, instrumental in determining the substrate specificity of catalytic subunits (2, 49). In addition, CK2β serves to amplify the kinase activity and ensures the stability of the holoenzyme (2). Structural predictions from AlphaFold 2 suggest that both Ckb1 and Ckb2 in *C. neoformans* share similarities with human CK2β. In particular*, C. neoformans* Ckb1 has extended disordered regions at the N- and C-termini than human CK2β. These structural differences between the N- and C-termini were also observed in Ckb1 of *C. albicans* and Ckb2 of *S. pombe*, which is phylogenetically closer to Ckb1 in *C. neoformans* than Ckb2 (Fig. S1B and S3H). Based on these observations, we hypothesize that these extended structural loops might hold key regulatory roles in *C. neoformans* Ckb1 that diverge from the known functions. In support of this, we observed that the *ckb1*∆ mutant exhibited a more severe growth defect at 37°C and abnormal cell cycle patterns than the *ckb2*∆ mutant. This suggested that these structural differences might play a unique role in the regulatory subunit of CK2 across fungal species, including *C. neoformans*, *C. albicans*, and *S. pombe*. Of note, our observations that the phenotypic defects in *ckb1*∆ *ckb2*∆ *cka1*∆ mutants surpassed those in *cka1*∆ mutants suggest that Ckb1 and Ckb2 may serve additional functions beyond their role in regulating Cka1. These intriguing aspects merit further exploration in upcoming research.

This study highlights the CK2 complex’s vital role in regulating growth and cell cycle in *C. neoformans*. While the specific mechanisms are yet to be fully understood, parallels exist with CK2’s role in *S. cerevisiae*, where it is essential for G1 and G2/M phase transitions and shows growth defects upon deletion of its catalytic subunits (50, 51). CK2 is known to modulate chromatin dynamics, such as nucleosome reassembly in *S. cerevisiae* and histone modifications in both budding yeast and humans (52, 53). Our data suggest that in *C. neoformans*, CK2 may influence growth and cell cycle through transcriptional and post-translational controls. Specifically, deletion of *CKA1* led to the upregulation and hyperphosphorylation of key transcriptional activators and chromatin modulators like Spt3, Gcn5, Ubp7, and Ubp10. This regulatory influence could account for CK2’s impact on nearly 10% of the *C. neoformans* genome.

Determining the upstream factors that regulate the CK2 complex in *C. neoformans* remains an open question. Published transcriptome data show that CK2 components are constitutively expressed across various growth conditions. In human models, CK2 regulation is multifaceted, involving intrinsic mechanisms like autophosphorylation and extrinsic factors that modify its kinase activity and stability (54, 55). External stimuli can include interactions with growth factors, CDKs, and immune cells, as well as small molecules like polyamines and miRNAs (54, 56–58). The proteasome and chaperone proteins like Hsp90 also influence CK2 levels and activity (59). In yeast models like *S. cerevisiae* and *C. albicans*, intricate regulatory networks involving CK2, Hsp90, and MAPK pathways like Hog1 have been observed (25, 60, 61). While our data suggest CK2’s role in regulating the Mpk1 and Hog1 MAPKs, calcineurin, and UPR pathways in *C. neoformans*, our proteomics and phosphoproteomics analyses did not identify an association between CK2 and either Hsp90 or TORC1, suggesting that other regulatory mechanisms may be operating. Further investigations are needed to elucidate these potential alternative pathways for CK2 regulation in *C. neoformans*.

CK2 is classically characterized as a serine/threonine kinase, yet emerging evidence, including our phosphoproteomics data, supports its capacity for dual specificity, extending to tyrosine phosphorylate tyrosine residues within *C. neoformans*. This aligns with observations in oncology, where elevated CK2 activity correlates with increased tyrosine phosphorylation of targeted proteins, implicating CK2’s kinase activity in such modifications (62). Notably, this phenomenon transcends cell types, observed in both mammalian and yeast cells, despite the latter’s paucity of tyrosine kinases. Historically, CK2’s role in the phosphorylation of a conserved tyrosine on histone H2A is well documented in both human cells and *S. cerevisiae*, where such activity is pivotal for transcriptional elongation (53). Moreover, proteins phosphorylated by CK2 are often implicated in cell cycle regulation and DNA replication (53, 63). In the context of *C. neoformans*, our findings suggest that the deletion of *CKA1* results in the phosphorylation of tyrosine residues on proteins associated with vesicle and cell cycle processes. Our data not only reinforce the conserved nature of CK2’s function across diverse organisms but also spotlight a nuanced layer of Cka1’s role in *C. neoformans*, potentially linked to essential cellular mechanisms such as cell cycle regulation and intracellular trafficking.

This study presents compelling evidence that the Cka1 kinase could serve as an effective therapeutic target for cryptococcosis. Key observations supporting this notion include the following: (1) Cka1 deletion impairs critical virulence factors such as thermotolerance, stress tolerance, and melanin/capsule production, (2) inhibition of Cka1 sensitizes the organisms to existing antifungal treatments, in contrast to *C. albicans* where *CKA2* deletion promotes fluconazole resistance (64), and (3) Cka1 deletion severely compromises the pathogen’s ability to cross the BBB, effectively nullifying its virulence. While the potential for cross-reactivity within human CK2α might raise concerns, our previous data revealed a 60,000-fold difference in the IC_50_ values of CX-4945, a CK2 inhibitor, between human CK2α and *C. neoformans* Cka1 (30), suggesting the feasibility of developing fungal-specific Cka1 inhibitors. In addition, given that cancer patients—often characterized by elevated CK2α levels, are particularly susceptible to fungal infections (65), drugs with dual anticancer and antifungal activities may offer synergistic benefits in specific clinical settings. Consequently, our next research avenue will prioritize the development of small molecules aimed at inhibiting cryptococcal Cka1, offering a promising therapeutic strategy for treating cryptococcosis.

## MATERIALS AND METHODS

### Strains and growth conditions

The strains used in this study are detailed in Data Set S1. *Cryptococcus neoformans* yeast cells were routinely cultured on a YPD plate containing 2% peptone, 1% yeast extract, 2% dextrose, and 2% Bacto agar at 30°C. For the selection of *C. neoformans* transformants generated through the biolistic particle delivery system, nourseothricin (100 µg/mL), G418 (50 µg/mL), or hygromycin B (150 µg/mL) was added to the YPD medium.

### Construction of mutant strains

We utilized the *C. neoformans* serotype A H99S (*MAT*α) strain to construct knockout mutants, employing split marker/double-joint (DJ) PCR methods (66, 67). Resistance markers for nourseothricin (nourseothricin acetyltransferase; *NAT*), neomycin/G418 (neomycin phosphotransferase; *NEO*), and hygromycin B (hygromycin B phosphotransferase; *HYG*) were integrated into gene disruption cassettes, as previously described (66). The primer sequences used are available in Data Set S1. First-round PCR amplified the 5′- and 3′- flanking regions of the targeted genes (*CKA1*, *CKB1*, and *CKB2*) using H99S genomic DNA as a template and specific primers L1/L2 and R1/R2. The *NAT*, *NEO*, and *HYG* markers were amplified from their respective plasmids (pNAT, pNEO, and pHYG) using M13Fe and M13Re primers. In the second round of overlap PCR, we used these first-round PCR products as templates to assemble gene disruption cassettes with primers L1/SM2 and SM1/R2 for *NAT*, L1/GSL and GSR/R2 for *NEO*, and L1/HSM2 and HSM1/R2 for *HYG*. For biolistic transformation, the H99S strain was cultured overnight in 50 mL of YPD medium at 30°C. Cells were collected and resuspended in 5 mL YPD. Around 200 µL of this suspension was spread onto YPD plates containing 1 M sorbitol and incubated for an additional 3 h at 30°C. Using a PDS-100 particle delivery system (Bio-Rad, USA), 0.6 µm gold microcarrier beads (Bio-Rad, USA) were coated with the PCR-amplified gene disruption cassettes. These were then biolistically introduced into cells. Post-transformation, cells were then incubated for 4 h at 30°C to restore membrane integrity and then transferred to the selection medium containing appropriate antibiotics.

### Construction of complemented and epitope/fluorescent protein-tagged strains

For constructing complemented strains, DNA fragments containing the promoter, terminator, and ORF of *CKA1*, *CKB1*, and *CKB2* were amplified and cloned into pTOP-V2 vectors. These constructs, designated pTOP_CKA1, pTOP_CKB1, and pTOP_CKB2, were verified by DNA sequencing. For non-tagging complemented strain, the inserts were subcloned into a pNEO vector to generate pNEO_CKA1, pNEO_CKB1, and pNEO_CKB2. For constructing *ckb1*Δ*::CKB1-mCherry* and *ckb2*Δ*::CKB2-mCherry* strains, the inserts were subcloned into pNEO_mCherryht plasmid, generating pNEO_CKB1-mCherryht and pNEO_CKB2-mCherryht. The linearized NdeI-digested pNEO_CKA1, NruI-digested pNEO_CKB1, and NruI-digested pNEO_CKB2 plasmids were introduced into *cka1*Δ (YSB3052), *ckb1*Δ (YSB6680), and *ckb2*Δ (YSB6727) strains, respectively. The linearized PpuMI-digested pNEO_CKB1-mCherryht and NruI-digested pNEO_CKB2-mCherryht were introduced in *ckb1*Δ and *ckb2*Δ strains, respectively. For constructing other mCherry-, 4xFLAG-, and 6xHA-tagged strains, the ORF without stop codon and promoter regions of *CKA1*, *CKB1*, and *CKB2* were PCR-amplified and cloned into pNEO-mCherryht, pNEO-4xFLAG, or pHYG-6xHA using Gibson Assembly Master Mix kit (New England BioLabs, USA). The plasmids pNEO_CKA1-4xFLAG (or CKA1-mCherrryht), pNEO_CKB1-4xFLAG, pHYG_CKB1-6xHA, and pHYG_CKB2-6xHA were linearized by digestion with NdeI for *CKA1*, PpuMI for *CKB1*, and NruI for *CKB2* and introduced in the *cka1*Δ, *ckb1*Δ, or *ckb2*Δ strain through biolistic transformation. Each cassette for C-terminal chromosomal tagging was generated using a split marker/double-joint PCR strategy to construct strains containing *CKB1-6xHA* or *CKB2-6xHA*. In the first round of PCR, the 3′-ORF region, 3′-flanking region, and the 4xHA-containing *HYG* region were all individually amplified. Then, the 3′-ORF region and 5′-*HYG*-split marker, and the 3′-flanking region and 3′-*HYG*-split marker were combined and used as templates in the second round of overlap PCR. Through biolistic transformation, the tagging cassettes were introduced into YSB7784 or YSB7812 strains. Diagnostic PCR and Southern blotting were employed to confirm targeted integration and genotype, respectively.

### Flow cytometry analysis

For cell preparation, wild-type and CK2 mutant strains were grown to an optical density at 600 nm (OD_600_) of 0.8, harvested, and washed with phosphate-buffered saline (PBS). For ethanol fixation, 10^6^ cells in 300 µL of PBS were gently mixed with 700 µL of 100% ethanol and incubated at 4°C for 16 h. Post-fixation, cells were washed with PBS containing 1% and 0.5% bovine serum albumin (BSA). Cells were then treated with 200 µg/mL RNase (#EN05331, Thermo Scientific, USA) for 30 min at 37°C. Following centrifugation, cells were stained with propidium iodide staining buffer [100 µg/mL propidium iodide, 100 mM Tris (pH 7.4), 150 mM NaCl, 1 mM CaCl_2_, 0.5 mM MgCl_2_, and 0.1% Nonidet P-40] for 2 h at room temperature in the dark. After a final PBS wash and filtration *via* a strainer, fluorescence was assessed using a BD FACS Symphony A5, capturing 10,000 events per sample.

### Scanning/transmission electron microscopy (SEM/TEM)

For sample preparation, cells with an OD_600_ of 0.8 were harvested and fixed in Karnovsky’s fixative (2% glutaraldehyde, 2% paraformaldehyde, 0.1 M phosphate buffer, pH 7.4) for 24 h, followed by two 30 min washes in 0.1 M phosphate buffer. For SEM, the specimens were post-fixed in 1% OsO_4_ for 2 h and then dehydrated using a Critical Point Dryer (EM CPD300, LEICA, Germany) with an ascending ethanol series (50%, 60%, 70%, 80%, 90%, 95%, and 100%). The samples were coated with a layer of platinum using ion sputtering (EM CPD300, LEICA, Germany) and subjected to observation using a field emission scanning electron microscope (MERLIN, ZEISS, Germany). For TEM, fixed cells were dehydrated with the ascending ethanol series for 10 min each and infiltrated with propylene oxide for 10 min. Specimens were embedded using a Poly/Bed 812 kit (Polysciences, USA), which was then polymerized in an electron microscope oven (TD-700, DOSAKA, Japan) at 65°C for 12 h. The block was sliced into 200 nm semi-thin sections with a diamond knife in Ultramichrome, dyed with toluidine blue for optical microscope testing. The area of interest was then cut into 80-nm-thin sections using an ultramicrotome, mounted on copper grids, double-stained with 3% uranyl acetate for 30 min and 3% lead citrated for 7 min, and imaged using an 80kV-acceleration transmission electron microscope (JEM-1011, JEOL, Japan) equipped with a Megaview III CCD camera (Soft imaging system, Germany).

### Visualizing the subcellular localization of mCherry-tagged proteins

Cka1-, Ckb1-, Ckb2-, and Crz1-mCherry-tagged strains were initially cultured overnight in YPD medium at 30°C. These cells were subcultured in YPD medium until reaching an OD_600_ of 0.8 and then fixed with a 4% paraformaldehyde solution containing 3.4% sucrose for 15 min at room temperature. After fixation, the cells were washed with a solution containing 0.1 M KPO_4_ and 1.2 M sorbitol and stained with 10 µg/mL Hoechst 33342 (Thermo Fisher, USA) for 30 min in the dark for nuclear visualization. The stained cells were then observed using both differential interference contrast (DIC) and fluorescence microscopy (Nikon Eclipse, Japan).

For visualizing the localization of Crz1-mCherry, P*_CRZ1_*-Crz1-mCherry (NEO) (JL334) (44), and P*_CRZ1_*-Crz1-mCherry (NEO) *cka1*Δ (YSB11149) strains were first cultured in YPD at 30°C. They were then diluted with fresh YPD media to an OD_600_ of 0.2 and incubated at 23°C until an OD_600_ of 0.8 was achieved. At this point, the cells were transferred to pre-warmed YPD broth at 37°C and incubated for an additional 30 min before following the same preparation and visualization procedures as other strains.

### Coimmunoprecipitation and western blotting

4xFLAG and 6xHA tagged strains were grown overnight in YPD medium at 30°C and subcultured in fresh YPD broth until reaching an OD_600_ of 0.8. Cells were then harvested, frozen in liquid nitrogen, and lysed using a lysis buffer containing 50 mM Tris-Cl (pH 7.5), 1% sodium deoxycholate, 5 mM NaF, 0.1% SDS, 1% Triton X-100, 0.5 mM phenylmethylsulfonyl fluoride, and 1× protease inhibitor cocktail solution (539136, Calbiochem, USA). After bead beating, the lysates were collected and incubated with an anti-FLAG antibody (F1804, Sigma-Aldrich, USA) overnight at 4°C. Sepharose protein G beads (17061801, GE Healthcare Life Sciences, USA) were added and incubated for another 6 h, then washed three times with the lysis buffer. Proteins bound to the beads were eluted with SDS sample buffer (50 mM Tris-Cl, 2% SDS, 10% glycerol, and 0.01% β-mercaptoethanol) and detected using anti-FLAG and anti-HA (sc-7392, Santa Cruz Biotechnology, USA) antibodies.

For tracking phosphorylation of Hog1 and Mpk1, each strain grown to OD_600_ of 0.8 as described above was exposed to a YPD medium containing 1 M NaCl or 1% CFW or shifted to pre-warmed YPD at 37°C. Cells were collected at different time points, rapidly frozen in liquid nitrogen, resuspended in the lysis buffer, and disrupted through repeated bead-beating. Proteins were quantified using the Pierce BCA Protein Assay Kit (23225, Thermo Scientific, USA), and equal amounts were run through a 10% SDS-PAGE gel. Blots were transferred onto PVDF membranes (Bio-Rad) and probed using antibodies specific for the phosphorylation forms of Hog1 [a phosphor-p38 MAPK antibody (#4511); Cell Signaling Technology, USA] and Mpk1 [Phospho-p44/42 MAPK (Erk1/2) antibody (#4370); Cell Signaling Technology, USA]. Monoclonal anti-β actin antibody (sc-47778, Santa Cruz Biotechnology, USA) and polyclonal anti-Hog1 antibody (custom made, AbFrontier, Republic of Korea) were used as loading controls. For the secondary antibody, a mouse anti-rabbit IgG peroxidase-conjugated antibody (sc-2357, Santa Cruz Biotechnology, USA) and mouse IgG Fc binding protein conjugated to horseradish peroxidase (sc-525409, Santa Cruz Biotechnology, USA) were used. The resulting blot was developed using chemiluminescence.

### *In vitro* phenotypic analysis

For examining the growth and chemical susceptibility, each strain was cultured in a YPD liquid medium for 16 h at 30°C. We then performed 10-fold serial dilutions (1 to 10^4^) and spotted them onto YPD plates with various chemical stressors. Plates were incubated at 30°C for up to 5 days and monitored daily with photos. Growth rates of the wild-type (H99S) and CK2 mutants were also compared using an automated multichannel bioreactor (RTS-8, Biosan Laboratories, Inc., USA) after overnight culture and adjusting the cell concentration to OD_600_ of 0.2. For assessing melanin production, overnight-cultured cells were washed twice in PBS and spotted onto Niger seed, dopamine, or epinephrine agar media (1 g L-asparagine, 3 g KH_2_PO_4_, 250 mg MgSO_4_, 1 mg thiamine, 5 g biotin, and 100 mg L-DOPA or epinephrine hydrochloride per L) containing 0.1% or 0.2% glucose. The plates were incubated at 37°C and imaged over 1 to 3 days. For capsule production assay, overnight cultured cells were spotted onto Littmans’s agar media after being washed with PBS, then incubated at 37°C. After 2 days, the cells were stained with India ink and observed under DIC microscopy. Capsule thickness was calculated based on 50 random cells, using the formula “Total diameter – cell body diameter.” Statistical significance was determined using one-way ANOVA with Bonferroni’s multiple-comparison test.

### Transcriptome analysis

The wild-type, *cka1*Δ, and *ckb1*Δ *ckb2*Δ *cka1*Δ strains were first cultured overnight at 30°C and then transferred to fresh YPD medium until they reached an OD_600_ of 0.8. Cells were collected, flash-frozen, and lyophilized. Total RNA was extracted using Easy-BLUE (17061, iNtRON, South Korea) and further purified with an RNeasy minikit (74106, Qiagen, Germany). This process was repeated for three independent cultures of each strain. Using 1 µg of total RNA, cDNA libraries were prepared with the TruSeq mRNA library kit (Illumina, USA) and sequenced on an Illumina platform.

Post-sequencing, reads were processed to remove adaptor and low-quality sequences using Cutadpat v2.4 with Python 3.7.4 with adapter sequence (68). The reads were processed as previously described (69) and aligned to the *C. neoformans* H99 reference genome using Hisat2 v2.2.1 and the Hisat and Bowtie2 algorithm. Annotation data were obtained from the NCBI FTP server. The “-p 30” and “—dta −1” options, along with other default settings, were used to run Hisat2. Aligned reads were converted and sorted using Samtools v0.1.19 with default parameters except for the “-Sb -@ 8” option for converting and the “-@ 20 –m 2000000000” option for sorting (70). The “-p 12” option was used by Stringtie v1.3.6 to perform transcript assembly and abundance estimation. Transcript abundance was quantified using FPKM (Fragments Per Kilobase of transcript per Million mapped reads) values (70). Data matrices were generated and analyzed using the R package “isoformswitchanalyzerR.” Quality control was assessed through DEBrowser (71). The analysis of differentially expressed genes (DEGs) was conducted with DESeq2 v1.24 (72, 73), and results were visualized using the Enhanced Volcano package in R v4.1.0 (74), with a cutoff of more than twofold changes and a *P*-value of less than 0.05.

### Proteomics and phosphoproteomics analysis

For sample preparation, the wild-type and *cka1*Δ mutant cells were cultured overnight at 30°C and transferred to fresh YPD medium until they reached OD_600_ of 0.8. The cells were collected, flash-frozen, resuspended in the lysis buffer, and lysed using a bead-beating method. Three sets of 300 µg whole cell lysates were subjected to the following processes. Briefly, proteins were reduced with 15 mM dithiothreitol for 1 h at 56°C and then alkylated with 60 mM iodoacetamide for 45 min at room temperature in the dark. Proteins were precipitated with TCA, digested overnight with trypsin solution (Promega, Madison, WI, USA) in a ratio of 1:50 (enzyme:protein) at 37°C, and dried. For quantitative analysis, digested proteins were subjected to isotope labeling with 18O water, incubated for 24 h at 37°C, and dried again. Peptides were then sorted using pH fractionation columns (Thermo Scientific, USA) with different pH elution buffers. The entire process was biologically replicated three times.

For nano-liquid chromatography (LC)-mass spectrometry and data analysis, 1 µg of the labeled peptides was prepared in 2 µL Solution A containing 0.1% formic acid for autosampler injection after the Zip Tip desalting process. These samples were loaded into nano-LC (Thermo Scientific, USA) connected to LTQ-Velos Orbitrap in the Mass Spectrometry Convergence Research Center at the Kyungpook National University. The solvents gradient of 3-23% of solution B (0.1% FA in ACN) was used in a high-performance liquid chromatography system (Ultimate 3000, Thermo Fisher Scientific, USA) equipped with an analytical column Acclaim PepMap RSLC C18 (75 µm × 500 mm, 2 µm particle, 100 Å pore size). Peptides were separated for 150 min at a flow rate 300 nL/min. The top 10 data-dependent mode was set to switch automatically between MS1 and MS2 acquisition in LTQ-Velos Orbitrap.

Data analysis was performed using the MaxQuant search engine (v.1.6) set against the *C. neoformans* H99 database. The following parameters were applied tryptic cleavage with two missed cleavage sites; C- termini for ^18^O labels; as well as fixed modification on Carbamidomethyl (C) and variable modification on acetyl (Protein N-term), Oxidation. This software was used for calculating ^18^O/^16^O ratios, allowing for accurate quantitative measurements of relative peptide/protein ratio. The false discovery rate for peptide spectrum matches (PSMs) was applied by 0.01. Differentially expressed proteins (DEPs) were identified based on a log_2_ fold change value of 1 or −1, and further bioinformatics analysis was carried out using Perseus (v. 1.6) to create a volcano plot. The correlation of triplicate was calculated by Pearson’s correlation (R).

### Quantitative RT-PCR for Hxl1 splicing

The wild-type and CK2 mutants were cultured overnight and then diluted to an OD_600_ of 0.2. The cultures were allowed to grow until they reached an OD_600_ of 0.8. At this point, the cultures were split into two sets: one treated with 5 µg/mL of tunicamycin and one that remained untreated. These were then incubated for an additional 2 days. The cells were collected, flash-frozen, and lyophilized. Total RNA was extracted following the method described above. For the cDNA synthesis, total RNA was adjusted to a concentration of 5 µg using DEPC-treated water, added with oligo(dT)-pdN6, and heated at 65°C for 5 min. Then the mixture was added with RNAse inhibitor (M007, Enzynomics, South Korea), dNTP, and reverse H minus Reverse transcriptase (EP0752, Thermo Scientific, USA) and incubated at 50°C for 60 min, followed by heat inactivation at 85°C for 10 min. To measure *HXL1* splicing, RT-PCR of *HXL1* and *ACT1* was performed with gene-specific primers (Data Set S1).

### Virulence assay using the murine model of systemic cryptococcosis

To assess the virulence of the wild-type and CK2 mutants, we employed a murine model using female BALB/c mice that were 7 to 8 weeks old (JA BIO, South Korea). Each strain was cultured overnight in YPD broth at 30°C, washed thrice with PBS, and adjusted to a concentration of 10^6^ cells/mL. Prior to the infection, each mouse was anesthetized using a blend of Avertin (2,2,2 tribrometylalcohol; Aldrich, USA), *tert-*amylalcohol (Aldrich, USA), and saline (Medion, South Korea). The mice were then infected through intranasal instillation, receiving a 20 µL cell suspension of 5 × 10^4^ cells. Post-infection, we monitored the health of the mice twice daily. Mice that showed extremes of morbidity, such as a 25% loss in body weight, the inability to eat or drink, or a slumped posture, were euthanized to minimize suffering. We used the log-rank (Mantel-Cox) test for statistical analysis of the survival data using Graph Pad Prism software version 8.0.1.

### *In vitro* BBB crossing assay

The BBB crossing assay was performed as previously described (35). Briefly, the human brain microvascular endothelial cell (HBMEC) line (hCMEC/D3, Merck, USA) was grown on collagen-precoated plates (354236, Corning, USA) and harvested when they reached 80% confluency. We then seeded 5 × 10^4^ hCMEC/D3 cells onto 8-μm-porous collagen-coated membranes (353097, BD Falcon) and cultured them in an EndoGRO^TM^-MV complete media kit (SCME004, Sigma-Aldrich, USA). The next day, we replaced the medium with fresh EndoGRO^TM^ medium supplemented with 2.5% human serum (H4522, Sigma, USA) and continued the cultivation for another 4 days. One day before yeast inoculation, we again changed the medium, this time to EndoGRO^TM^ medium supplemented with 1.25% human serum. The cells were kept in a 5% CO_2_ incubator at 37°C. The trans-endothelial electrical resistance (TEER) was measured using an Epithelial Volt per Ohm Meter (EVOM^2^ device, World Precision Instrument, USA) to test the integrity of tight junctions between hCMEC/D3 cells. Overnight cultured wild-type and CK2 mutant cells were washed thrice with PBS and adjusted to a concentration of 5 × 10^5^ cells in 100 µL PBS. The yeast cells were added to the top of the hCMEC/D3-seeded porous membrane. After a 24-h incubation at 37°C and 5% CO_2_ conditions, we collected the yeast cells that had passed through the membrane. The BBB crossing ratio for each mutant strain was normalized to the wild-type strain to obtain the relative BBB crossing ratio.

## Data Availability

All strains and materials utilized in this study are available upon request. Transcriptome profiling data from RNA sequencing, covering wild-type, *cka1*Δ, and *ckb1*Δ *ckb2*Δ *cka1*Δ strains, have been submitted to the Gene Expression Omnibus and can be accessed with the accession number GSE245849. Proteomics and phosphoproteomics profiling data have been deposited to the ProteomeXchange Consortium through the PRIDE (75) partner repository and are available under the data set identifier PXD033626.

## SUPPLEMENTAL MATERIAL

Data Set S1 mbio.03275-23-s0001.xlsx *C. neoformans* strains and primers used in this study.

Data Set S2 mbio.03275-23-s0002.xlsx Differential expression fold change scores from the RNA sequencing-based transcriptome analysis of CK2 mutants in *C. neoformans*.

Data Set S3 mbio.03275-23-s0003.xlsx Proteomic and phosphoproteomic fold.change scores from the comparative analysis of CK2 mutants in *C. neoformans*.

Fig. S1 mbio.03275-23-s0004.pdf Comparative phylogenetic analysis of CK2 subunit orthologs across human and fungal species.

Fig. S2 mbio.03275-23-s0005.pdf Generation and verification of CK2 subunit deleted strains and complemented strains.

Fig. S3 mbio.03275-23-s0006.pdf Generation and validation of epitope-tagged CK2 strains.

Fig. S4 mbio.03275-23-s0007.pdf Generation and validation of mCherry-tagged CK2 strains.

Fig. S5 mbio.03275-23-s0008.pdf The role of CK2 in stress adaptation and virulence in *C. neoformans*.

Fig. S6 mbio.03275-23-s0009.pdf Transcriptomic analysis and detailed proteomic and phosphoproteomic analysis overview.

Fig. S7 mbio.03275-23-s0010.pdf Generation and characterization of the *cka1* mutant expressing Crz1-mCherry.

ASM does not own the copyrights to Supplemental Material that may be linked to, or accessed through, an article. The authors have granted ASM a non-exclusive, world-wide license to publish the Supplemental Material files. Please contact the corresponding author directly for reuse.
