# Supplementary material for: Casein kinase 2 complex: a central regulator of multiple pathobiological signaling pathways in Cryptococcus neoformans
Source: mBio. 2024 Jan 9;15(2):e03275-23. doi: 10.1128/mbio.03275-23 (PMC10865844; doi:10.1128/mbio.03275-23)
Supplement: Fig. S1 — Comparative phylogenetic analysis of CK2 subunit orthologs across human and fungal species. [file mbio.03275-23-s0004.pdf]

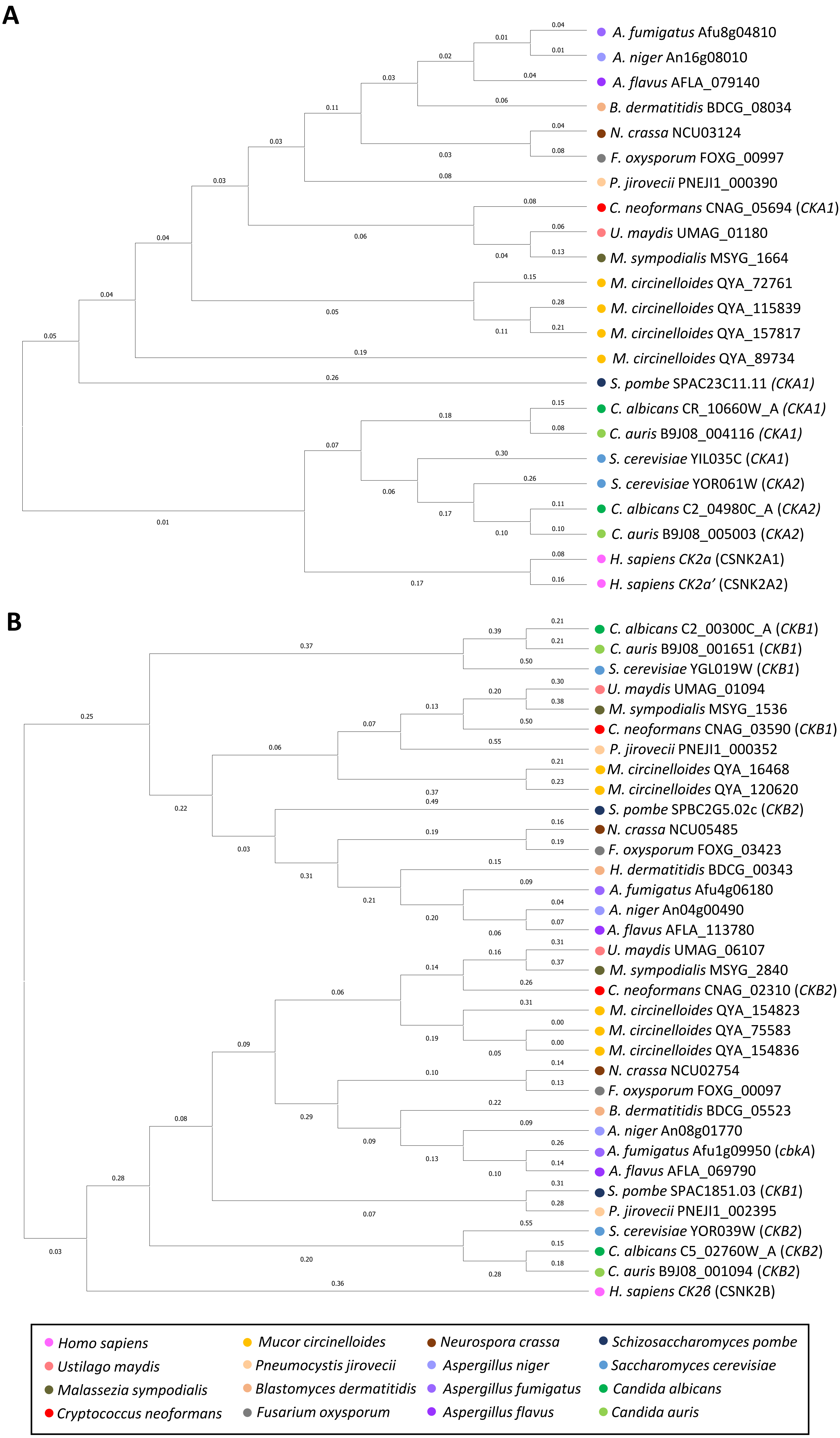
A
A. fumigatus Afu8g04810
A. niger An16g08010
A. flavus AFLA_079140
B. dermatitidis BDCG_08034
N. crassa NCU03124
F. oxysporum FOXG_00997
P. jirovecii PNEJI1_000390
C. neoformans CNAG_05694 (CKA1)
U. maydis UMAG_01180
M. sympodialis MSYG_1664
M. circinelloides QYA_72761
M. circinelloides QYA_115839
M. circinelloides QYA_157817
M. circinelloides QYA_89734
S. pombe SPAC23C11.11 (CKA1)
C. albicans CR_10660W_A (CKA1)
C. auris B9J08_004116 (CKA1)
S. cerevisiae YIL035C (CKA1)
S. cerevisiae YOR061W (CKA2)
C. albicans C2_04980C_A (CKA2)
C. auris B9J08_005003 (CKA2)
H. sapiens CK2a (CSNK2A1)
H. sapiens CK2a' (CSNK2A2)
B
C. albicans C2_00300C_A (CKB1)
C. auris B9J08_001651 (CKB1)
S. cerevisiae YGL019W (CKB1)
U. maydis UMAG_01094
M. sympodialis MSYG_1536
C. neoformans CNAG_03590 (CKB1)
P. jirovecii PNEJI1_000352
M. circinelloides QYA_16468
M. circinelloides QYA_120620
S. pombe SPBC2G5.02c (CKB2)
N. crassa NCU05485
F. oxysporum FOXG_03423
H. dermatitidis BDCG_00343
A. fumigatus Afu4g06180
A. niger An04g00490
A. flavus AFLA_113780
U. maydis UMAG_06107
M. sympodialis MSYG_2840
C. neoformans CNAG_02310 (CKB2)
M. circinelloides QYA_154823
M. circinelloides QYA_75583
M. circinelloides QYA_154836
N. crassa NCU02754
F. oxysporum FOXG_00097
B. dermatitidis BDCG_05523
A. niger An08g01770
A. fumigatus Afu1g09950 (cbkA)
A. flavus AFLA_069790
S. pombe SPAC1851.03 (CKB1)
P. jirovecii PNEJI1_002395
S. cerevisiae YOR039W (CKB2)
C. albicans C5_02760W_A (CKB2)
C. auris B9J08_001094 (CKB2)
H. sapiens CK2β (CSNK2B)
Homo sapiens
Ustilago maydis
Malassezia sympodialis
Cryptococcus neoformans
Mucor circinelloides
Pneumocystis jirovecii
Blastomyces dermatitidis
Fusarium oxysporum
Neurospora crassa
Aspergillus niger
Aspergillus fumigatus
Aspergillus flavus
Schizosaccharomyces pombe
Saccharomyces cerevisiae
Candida albicans
Candida auris

FIG S1. Comparative phylogenetic analysis of CK2 subunit orthologs across human and fungal species. (A, B) Constructed phylogenetic trees represent the catalytic (A) and regulatory (B) subunit orthologs of the CK2 complex. The required protein sequences were sourced from FungiDB (https://fungidb.org). MEGA11 was employed to conduct evolutionary analysis.
