# Supplementary material for: Casein kinase 2 complex: a central regulator of multiple pathobiological signaling pathways in Cryptococcus neoformans
Source: mBio. 2024 Jan 9;15(2):e03275-23. doi: 10.1128/mbio.03275-23 (PMC10865844; doi:10.1128/mbio.03275-23)
Supplement: Fig. S2 — Generation and verification of CK2 subunit deleted strains and complemented strains. [file mbio.03275-23-s0005.pdf]

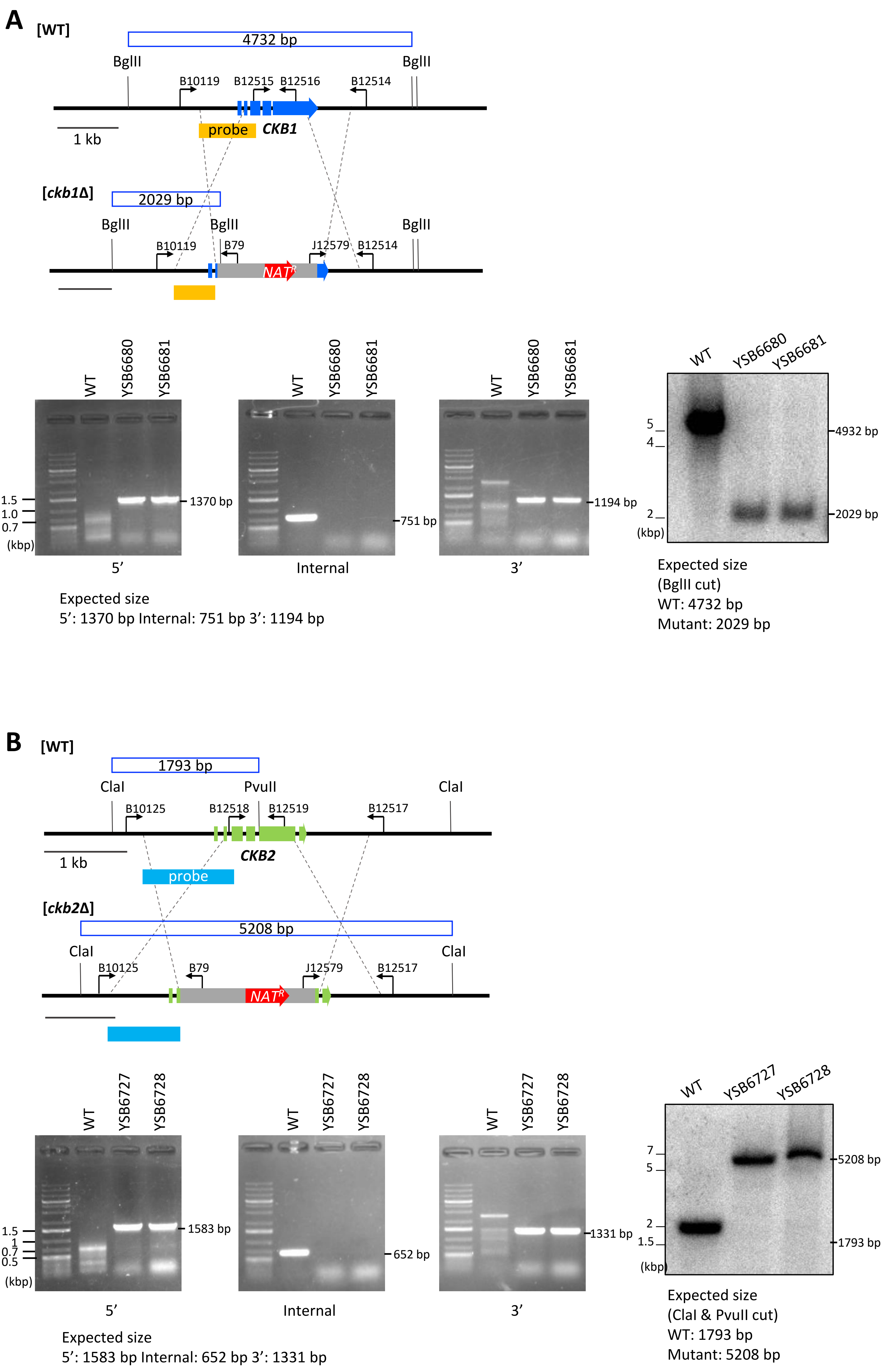
A
[WT]
4732 bp
BglII
BglII
B10119
B12515
B12516
B12514
1 kb
probe
CKB1
[ckb1Δ]
2029 bp
BglII
BglII
BglII
B10119
B79
J12579
B12514
NAT^R
WT
YSB6680
YSB6681
1.5
1.0
0.7
(kbp)
1370 bp
5'
WT
YSB6680
YSB6681
751 bp
Internal
WT
YSB6680
YSB6681
1194 bp
3'
Expected size
5': 1370 bp Internal: 751 bp 3': 1194 bp
WT
YSB6680
YSB6681
5
4
2
(kbp)
4932 bp
2029 bp
Expected size
(BglII cut)
WT: 4732 bp
Mutant: 2029 bp
B
[WT]
1793 bp
ClaI
PvuII
ClaI
B10125
B12518
B12519
B12517
1 kb
CKB2
probe
[ckb2Δ]
5208 bp
ClaI
ClaI
B10125
B79
J12579
B12517
NAT^R
WT
YSB6727
YSB6728
1.5
1
0.7
0.5
(kbp)
1583 bp
5'
WT
YSB6727
YSB6728
652 bp
Internal
WT
YSB6727
YSB6728
1331 bp
3'
Expected size
5': 1583 bp Internal: 652 bp 3': 1331 bp
WT
YSB6727
YSB6728
7
5
2
1.5
(kbp)
5208 bp
1793 bp
Expected size
(ClaI & PvuII cut)
WT: 1793 bp
Mutant: 5208 bp

C

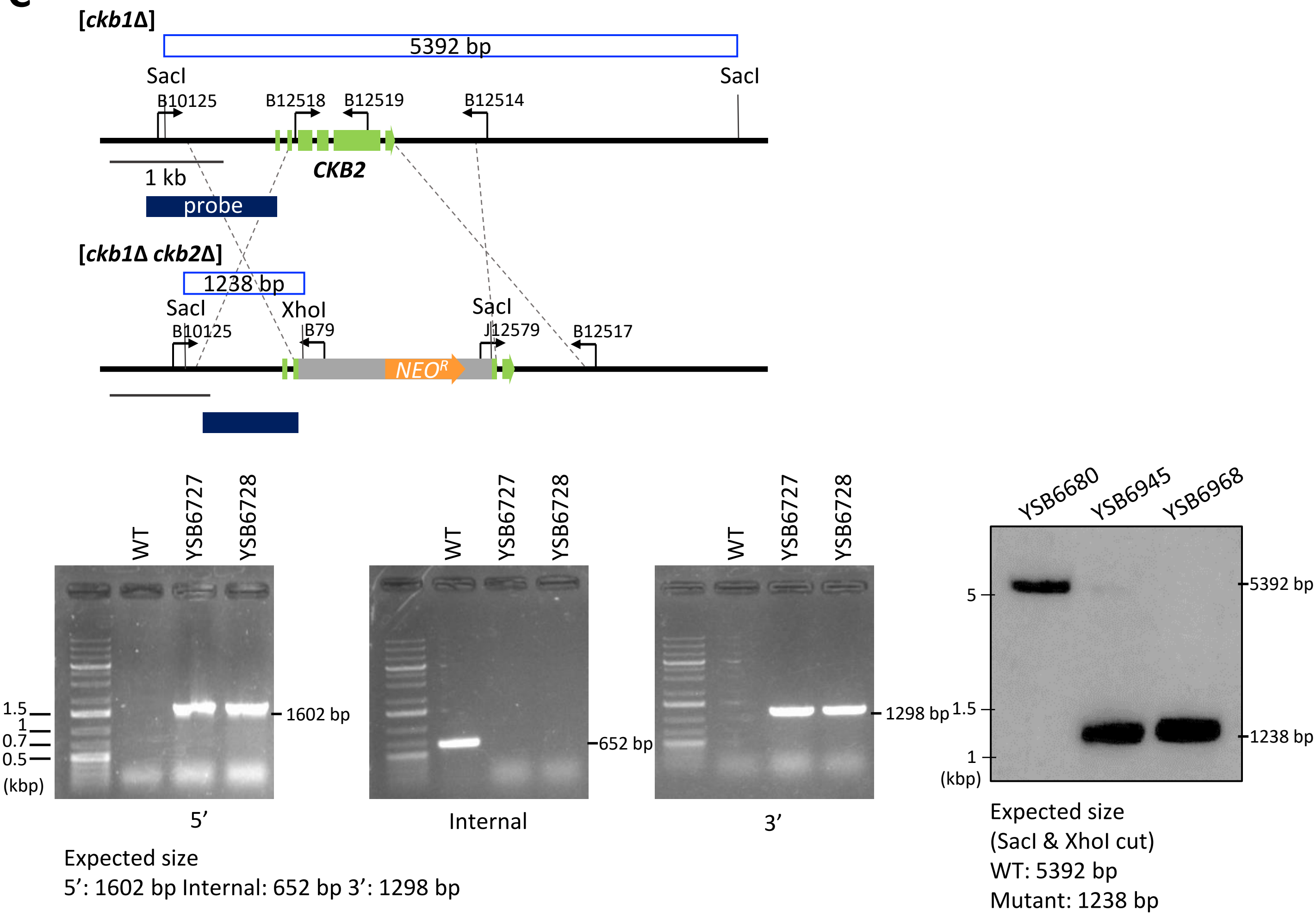
[ckb1Δ]
5392 bp
SacI
SacI
B10125
B12518
B12519
B12514
CKB2
1 kb
probe
[ckb1Δ ckb2Δ]
1238 bp
SacI
XhoI
SacI
B10125
B79
J12579
B12517
NEO^R
WT
YSB6727
YSB6728
1.5
1
0.7
0.5
(kbp)
1602 bp
5'
652 bp
Internal
1298 bp
3'
Expected size
5': 1602 bp Internal: 652 bp 3': 1298 bp
YSB6680
YSB6945
YSB6968
5
1.5
1
(kbp)
5392 bp
1238 bp
Expected size
(SacI & XhoI cut)
WT: 5392 bp
Mutant: 1238 bp

D

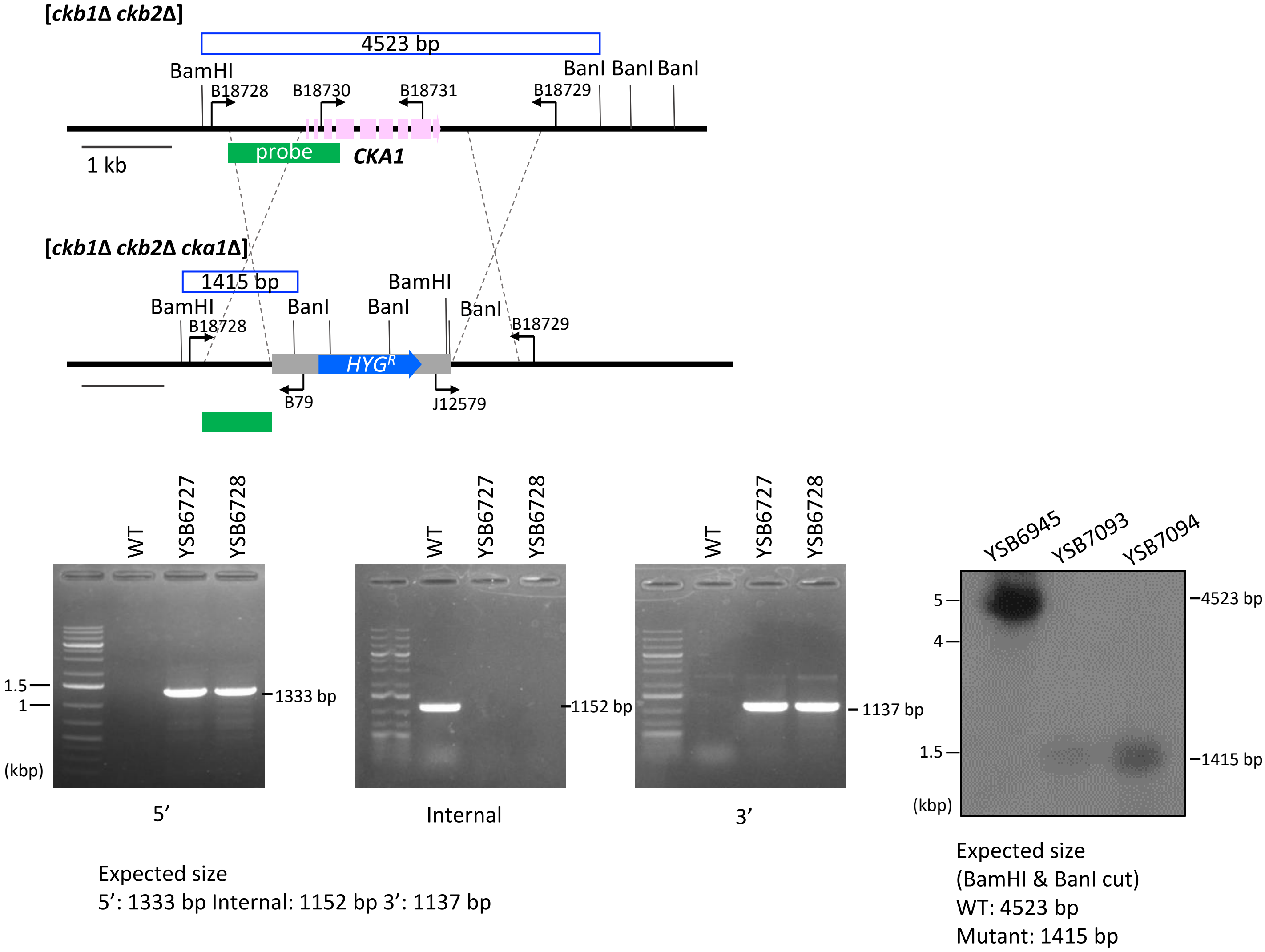
[ckb1Δ ckb2Δ]
4523 bp
BamHI
BanI BanI BanI
B18728
B18730
B18731
B18729
1 kb
probe
CKA1
[ckb1Δ ckb2Δ cka1Δ]
1415 bp
BamHI
BanI
BanI
BamHI
BanI
B18728
B18729
HYG^R
B79
J12579
WT
YSB6727
YSB6728
1.5
1
(kbp)
1333 bp
5'
1152 bp
Internal
1137 bp
3'
Expected size
5': 1333 bp Internal: 1152 bp 3': 1137 bp
YSB6945
YSB7093
YSB7094
5
4
1.5
(kbp)
4523 bp
1415 bp
Expected size
(BamHI & BanI cut)
WT: 4523 bp
Mutant: 1415 bp

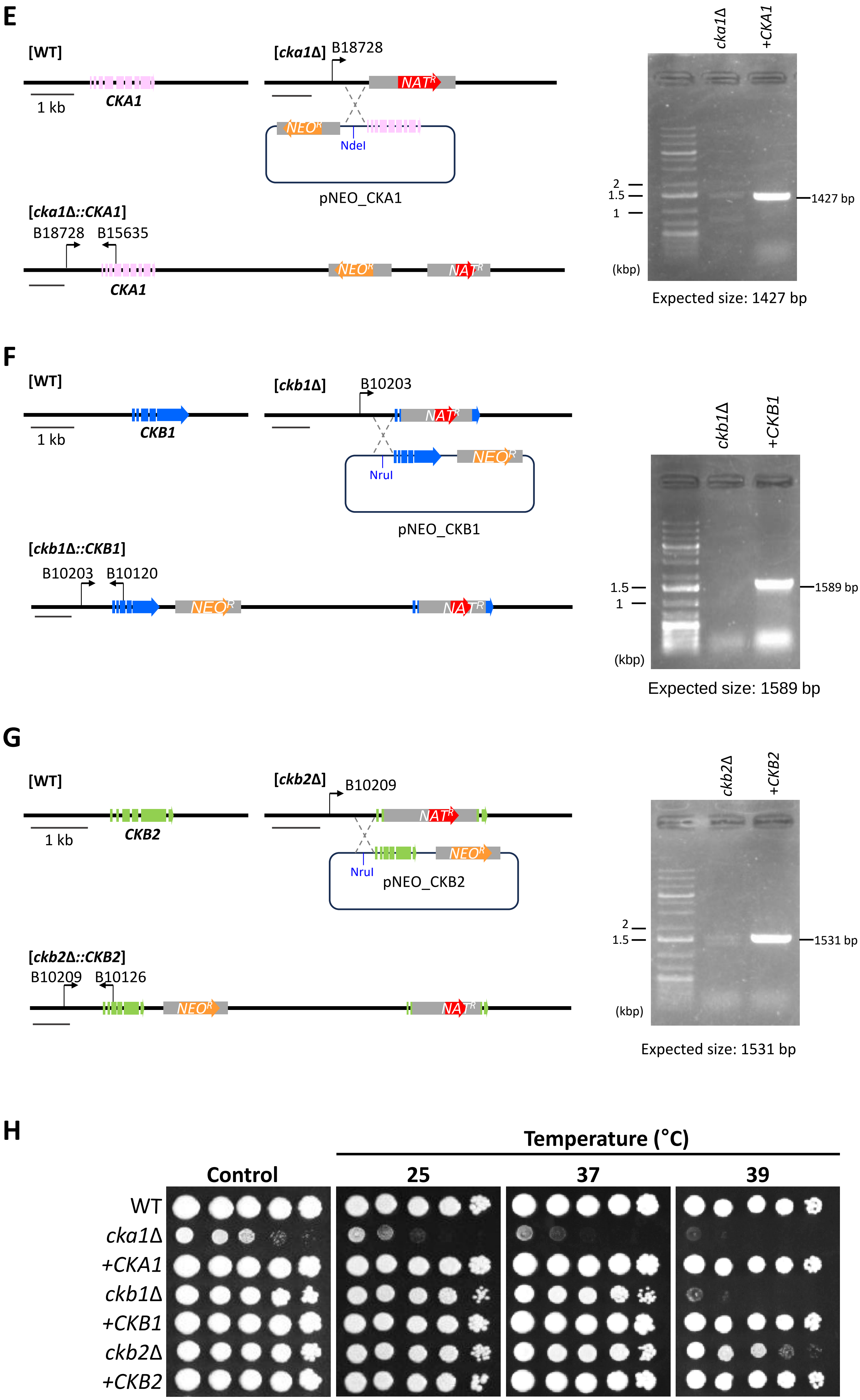

E
[WT]
1 kb
CKA1
[cka1Δ]
B18728
NAT^R
NEO^R
NdeI
pNEO_CKA1
[cka1Δ::CKA1]
B18728
B15635
CKA1
NEO^R
NAT^R
cka1Δ
+CKA1
2
1.5
1
(kbp)
1427 bp
Expected size: 1427 bp
F
[WT]
1 kb
CKB1
[ckb1Δ]
B10203
NAT^R
NruI
NEO^R
pNEO_CKB1
[ckb1Δ::CKB1]
B10203
B10120
NEO^R
NAT^R
ckb1Δ
+CKB1
1.5
1
(kbp)
1589 bp
Expected size: 1589 bp
G
[WT]
1 kb
CKB2
[ckb2Δ]
B10209
NAT^R
NruI
NEO^R
pNEO_CKB2
[ckb2Δ::CKB2]
B10209
B10126
NEO^R
NAT^R
ckb2Δ
+CKB2
2
1.5
(kbp)
1531 bp
Expected size: 1531 bp
H
Temperature (°C)
Control
25
37
39
WT
cka1Δ
+CKA1
ckb1Δ
+CKB1
ckb2Δ
+CKB2

I

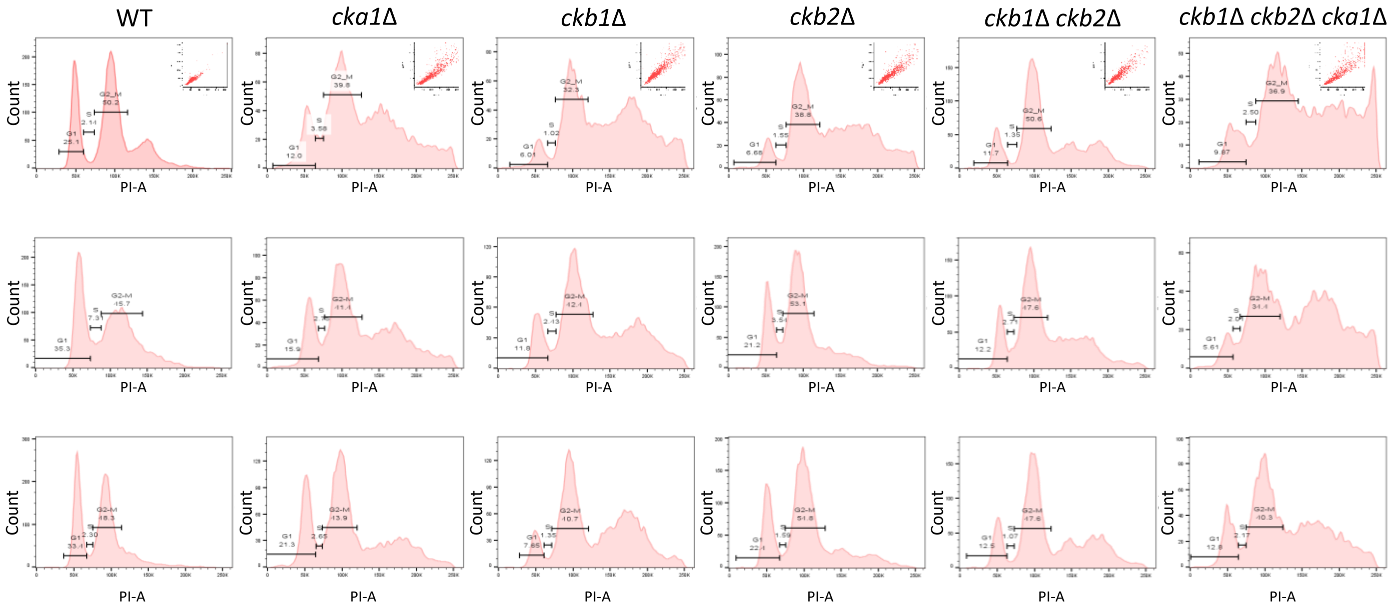

FIG S2. Generation and verification of CK2 subunit deleted strains and complemented strains. (A-D) Genotypic characterization of *ckb1*Δ, *ckb2*Δ, *ckb1*Δ *ckb2*Δ, and *ckb1*Δ *ckb2*Δ *cka1*Δ mutants is presented. The upper panel depicts the gene disruption strategy where target genes are supplanted by *NAT*-, *NEO*-, or *HYG*- selection markers. The lower panel showcases diagnostic PCR results, confirming 5'-end and 3'-end recombination events and internal deletion of individual target genes. Further validation through Southern blot is given on the right panel: (A) Genomic DNA from wild-type (H99S) and *ckb1*Δ mutants (YSB6680 and YSB6681) underwent BglII digestion. (B) Genomic DNA from wild-type (H99S) and *ckb2*Δ mutants (YSB6727 and YSB6728) was processed using ClaI and PvuII. (C) Genomic DNA from *ckb1*Δ (YSB6680) and *ckb1*Δ *ckb2*Δ mutants (YSB6945 and YSB6968) was subjected to SacI and XhoI digestion. (D) Genomic DNA from *ckb1*Δ *ckb2*Δ (YSB6945) and *ckb1*Δ *ckb2*Δ *cka1*Δ mutants (YSB7093 and YSB7094) was digested using BamHI and BanI. (E, F, and G) For the construction of complemented strains, individual DNA sequences, inclusive of the native promoter and terminator of *CKA1* (E), *CKB1* (F), and *CKB2* (G), were subcloned into the pNEO vector. These assembled plasmids were linearized using specific restriction enzymes, as outlined in the Materials and Methods. They were then introduced into their respective deletion mutants: *cka1*Δ (YSB3052), *ckb1*Δ (YSB6680), and *ckb2*Δ (YSB6727). Diagnostic PCR, employing primer pairs mentioned in Data Set S1, confirmed the precise integration of each plasmid. (H) The growth patterns of various strains - WT (H99S), *cka1*Δ (YSB3052), *cka1*Δ::*CKA1* (YSB6746), *ckb1*Δ (YSB6680), *ckb1*Δ::*CKB1* (YSB6840), *ckb2*Δ (YSB6727), and *ckb2*Δ::*CKB2* (YSB6826) – were examined. After overnight culturing at 30°C in YPD broth, they underwent 10-fold serial dilutions and were subsequently spotted onto YPD plates. These plates were then incubated across temperatures of 25°C, 30°C, 37°C, and 39°C for a duration of 4 days. (I) Cell cycle analysis of CK2 mutants via flow cytometry. Cells were stained with propidium iodide (PI), and for each sample, 10,000 cells were analyzed. The provided figures represent data from three biological replicates, organized in a top-to-bottom sequence. In each graph, the distribution percentages for different cell cycle phases – G1, S, and G2/M – are indicated by their respective DNA content levels.
