# Supplementary material for: Casein kinase 2 complex: a central regulator of multiple pathobiological signaling pathways in Cryptococcus neoformans
Source: mBio. 2024 Jan 9;15(2):e03275-23. doi: 10.1128/mbio.03275-23 (PMC10865844; doi:10.1128/mbio.03275-23)
Supplement: Fig. S3 — Generation and validation of epitope-tagged CK2 strains. [file mbio.03275-23-s0006.pdf]

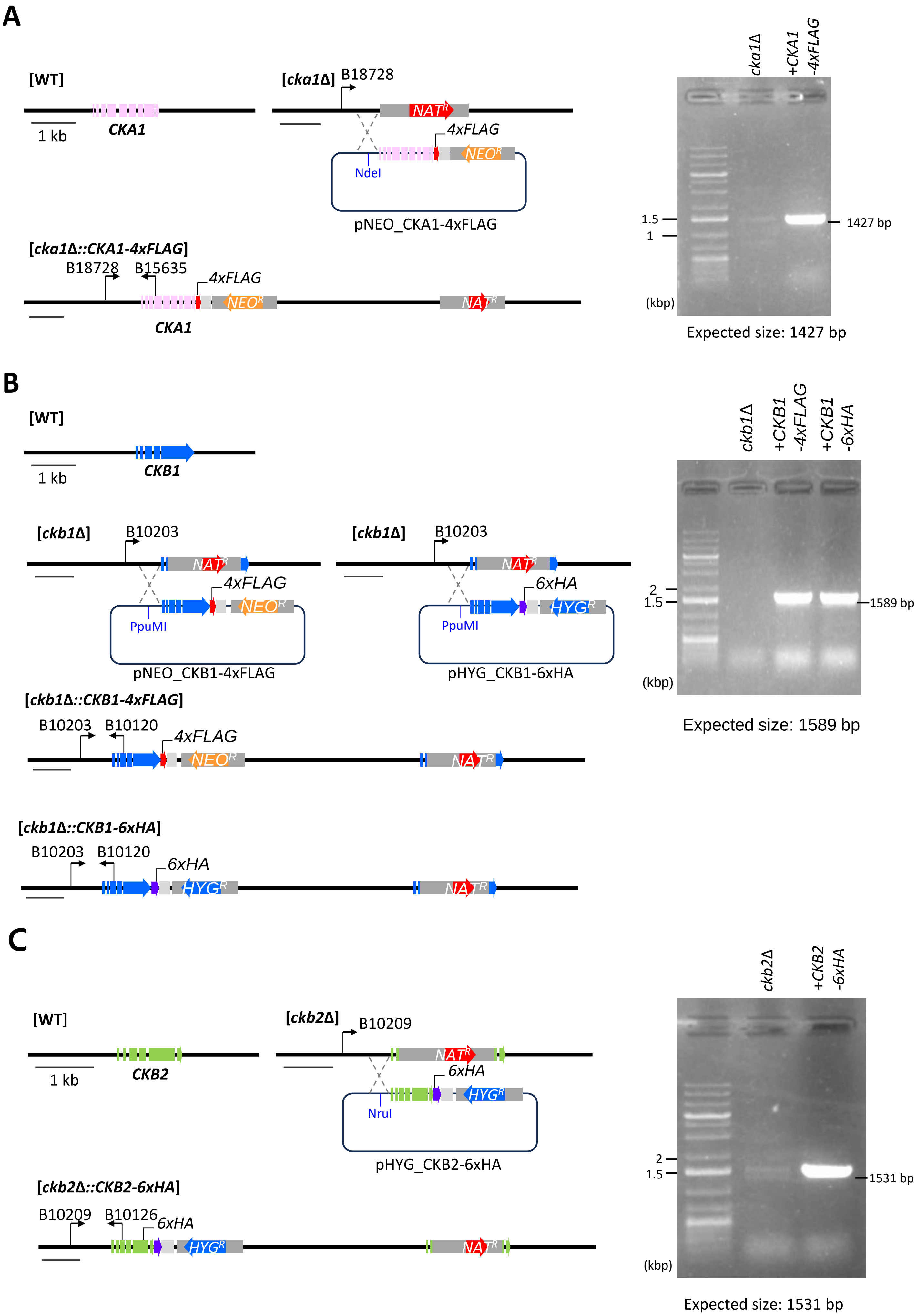
A
[WT]
1 kb
CKA1
[cka1Δ]
B18728
NAT^R
4xFLAG
NEO^R
NdeI
pNEO_CKA1-4xFLAG
[cka1Δ::CKA1-4xFLAG]
B18728
B15635
4xFLAG
NEO^R
NAT^R
CKA1
cka1Δ
+CKA1 -4xFLAG
1.5
1
(kbp)
1427 bp
Expected size: 1427 bp
B
[WT]
1 kb
CKB1
[ckb1Δ]
B10203
NAT^R
4xFLAG
NEO^R
PpuMI
pNEO_CKB1-4xFLAG
[ckb1Δ]
B10203
NAT^R
6xHA
HYG^R
PpuMI
pHYG_CKB1-6xHA
[ckb1Δ::CKB1-4xFLAG]
B10203
B10120
4xFLAG
NEO^R
NAT^R
[ckb1Δ::CKB1-6xHA]
B10203
B10120
6xHA
HYG^R
NAT^R
ckb1Δ
+CKB1 -4xFLAG
+CKB1 -6xHA
2
1.5
(kbp)
1589 bp
Expected size: 1589 bp
C
[WT]
1 kb
CKB2
[ckb2Δ]
B10209
NAT^R
6xHA
HYG^R
NruI
pHYG_CKB2-6xHA
[ckb2Δ::CKB2-6xHA]
B10209
B10126
6xHA
HYG^R
NAT^R
ckb2Δ
+CKB2 -6xHA
2
1.5
(kbp)
1531 bp
Expected size: 1531 bp

D

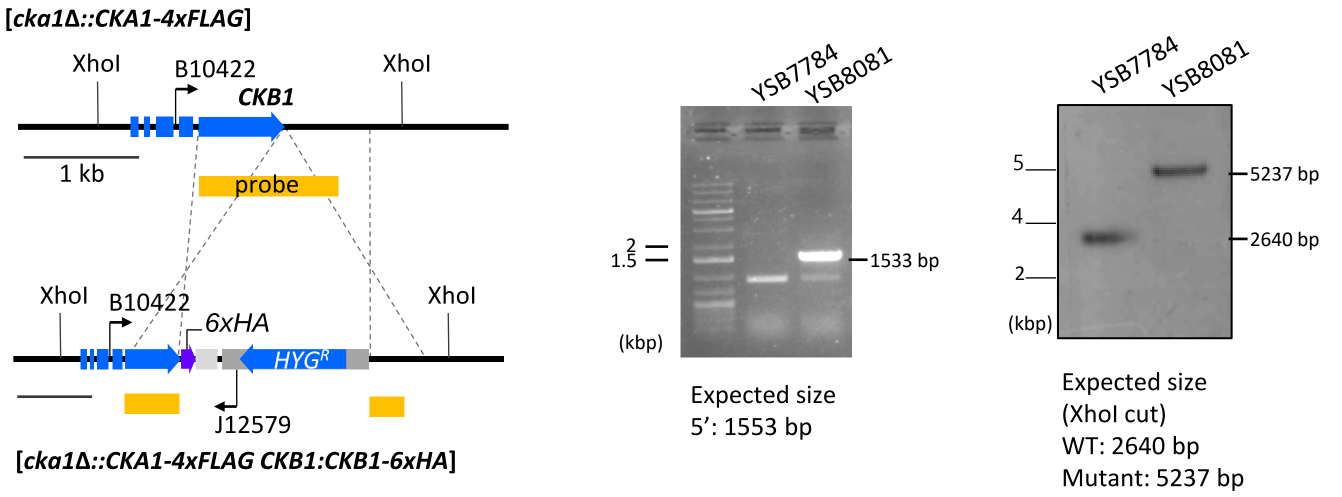
[cka1Δ::CKA1-4xFLAG]
XhoI
B10422
CKB1
XhoI
1 kb
probe
XhoI
B10422
6xHA
XhoI
HYG^R
J12579
[cka1Δ::CKA1-4xFLAG CKB1:CKB1-6xHA]
YSB7784
YSB8081
2
1.5
(kbp)
1533 bp
Expected size
5′: 1553 bp
YSB7784
YSB8081
5
4
2
(kbp)
5237 bp
2640 bp
Expected size
(XhoI cut)
WT: 2640 bp
Mutant: 5237 bp

E

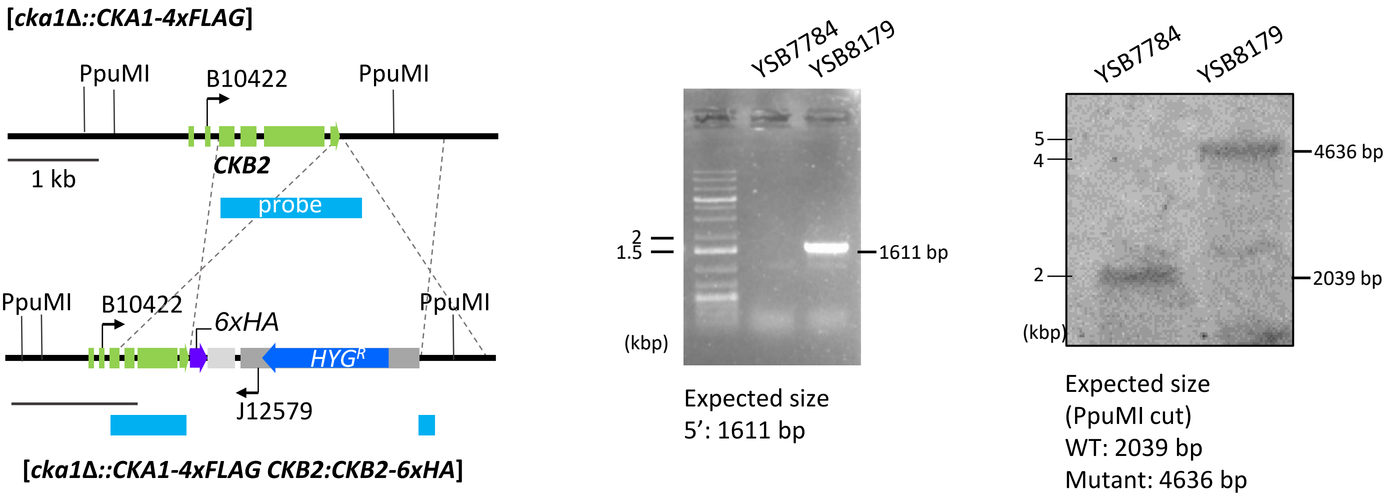
[cka1Δ::CKA1-4xFLAG]
PpuMI
B10422
PpuMI
1 kb
CKB2
probe
PpuMI
B10422
6xHA
PpuMI
HYG^R
J12579
[cka1Δ::CKA1-4xFLAG CKB2:CKB2-6xHA]
YSB7784
YSB8179
2
1.5
(kbp)
1611 bp
Expected size
5′: 1611 bp
YSB7784
YSB8179
5
4
2
(kbp)
4636 bp
2039 bp
Expected size
(PpuMI cut)
WT: 2039 bp
Mutant: 4636 bp

F

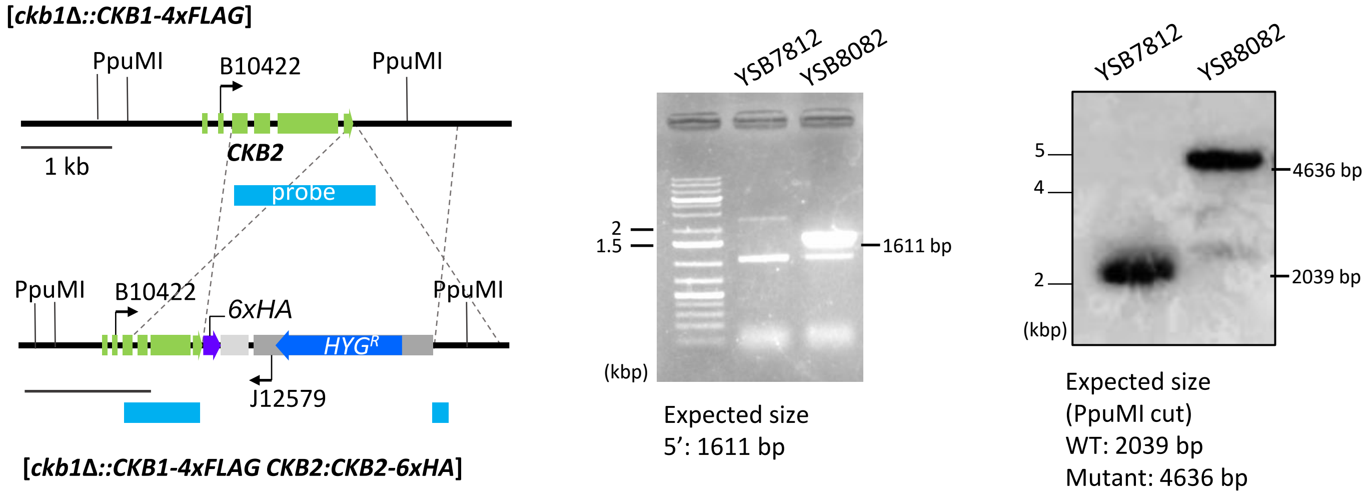
[ckb1Δ::CKB1-4xFLAG]
PpuMI
B10422
PpuMI
1 kb
CKB2
probe
PpuMI
B10422
6xHA
PpuMI
HYG^R
J12579
[ckb1Δ::CKB1-4xFLAG CKB2:CKB2-6xHA]
YSB7812
YSB8082
2
1.5
(kbp)
1611 bp
Expected size
5′: 1611 bp
YSB7812
YSB8082
5
4
2
(kbp)
4636 bp
2039 bp
Expected size
(PpuMI cut)
WT: 2039 bp
Mutant: 4636 bp

G

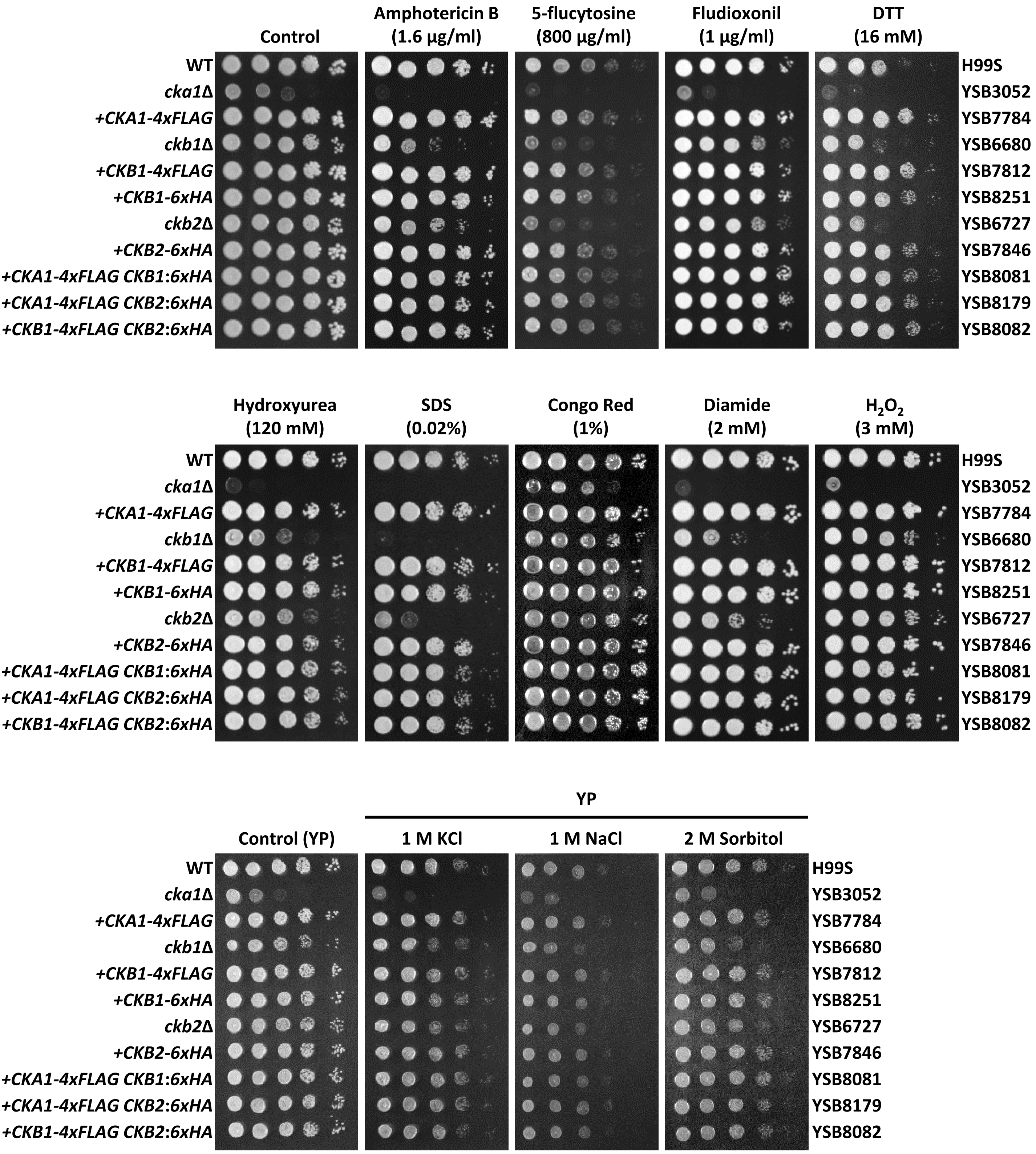

Control
Amphotericin B (1.6 µg/ml)
5-flucytosine (800 µg/ml)
Fludioxonil (1 µg/ml)
DTT (16 mM)
WT
cka1Δ
+CKA1-4xFLAG
ckb1Δ
+CKB1-4xFLAG
+CKB1-6xHA
ckb2Δ
+CKB2-6xHA
+CKA1-4xFLAG CKB1:6xHA
+CKA1-4xFLAG CKB2:6xHA
+CKB1-4xFLAG CKB2:6xHA
H99S
YSB3052
YSB7784
YSB6680
YSB7812
YSB8251
YSB6727
YSB7846
YSB8081
YSB8179
YSB8082
Hydroxyurea (120 mM)
SDS (0.02%)
Congo Red (1%)
Diamide (2 mM)
H_2O_2 (3 mM)
YP
Control (YP)
1 M KCl
1 M NaCl
2 M Sorbitol

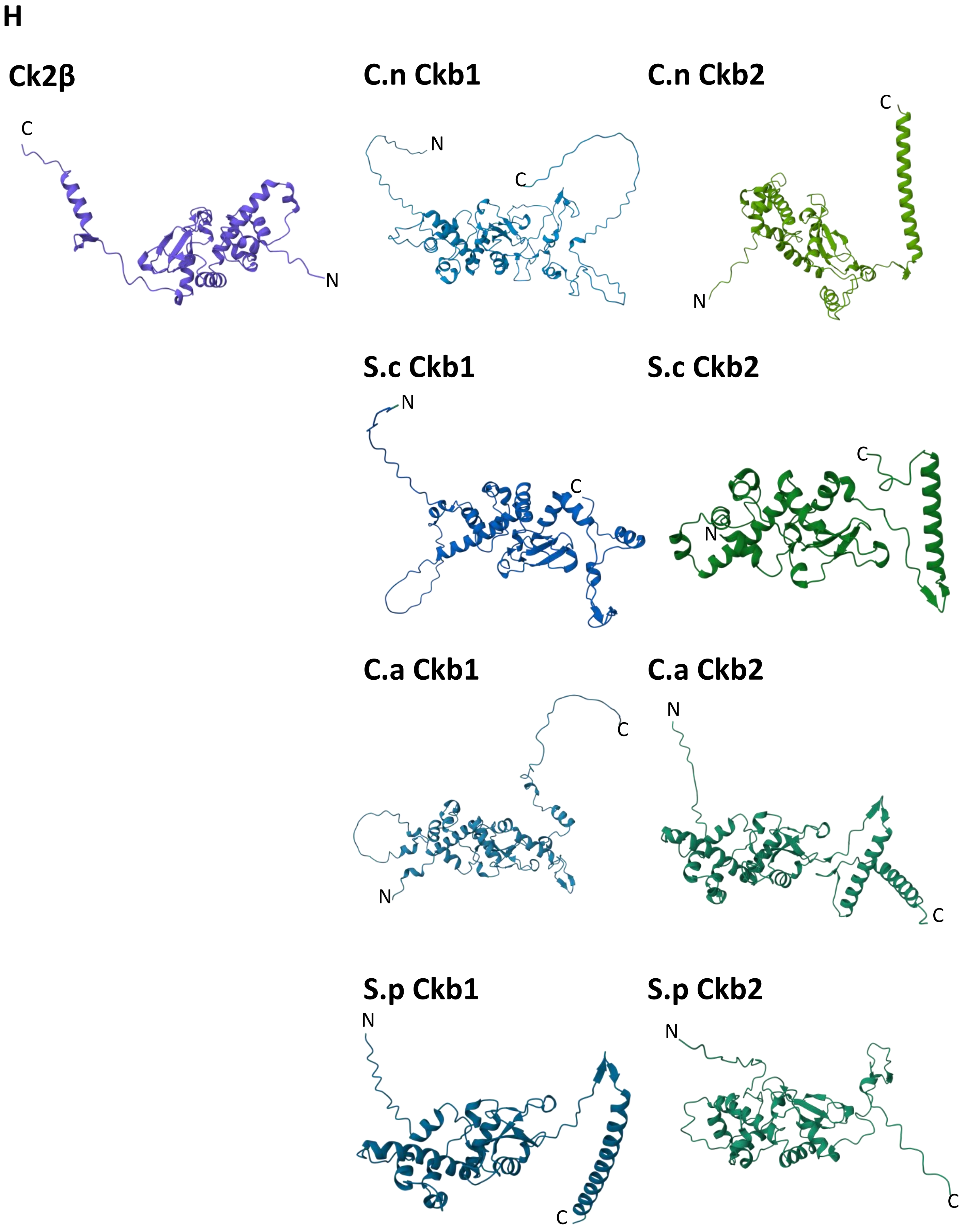
H
Ck2β
C
N
C.n Ckb1
N
C
C.n Ckb2
C
N
S.c Ckb1
N
C
S.c Ckb2
C
N
C.a Ckb1
C
N
C.a Ckb2
N
C
S.p Ckb1
N
C
S.p Ckb2
N
C

FIG S3. Generation and validation of epitope-tagged CK2 strains and structural prediction of CK2 regulatory subunits. (A-C) For each strain, the DNA sequence encompassing the promoter and ORF of *CKA1* (A), *CKB1* (B), and *CKB2* (C) was cloned into the pNEO_4xFLAG or pNEO_6xHA vector. Following linearization with specific restriction enzymes, as described in the Materials and Methods, these plasmids were biolistically introduced into corresponding deletion mutants: *cka1*Δ (YSB3052), *ckb1*Δ (YSB6680), and *ckb2*Δ (YSB6727). The successful targeted integration was authenticated using diagnostic PCR, utilizing the primers specified in the Data Set S1. (D) The C-terminus of Ckb1 in the *cka1*Δ::*CKA1-4xFLAG-NEO* strain (YSB7784) was tagged with 6xHA. Its correction insertion was verified using both diagnostic PCR and Southern blot analysis. (E, F) The C-terminus of Ckb2 in strains *cka1*Δ::*CKA1-4xFLAG-NEO* (YSB7784) and *ckb1*Δ::*CKB1-4xFLAG-NEO* (YSB7812) was tagged with 6xHA. The successful 5'-end recombination was confirmed by diagnostic PCR and Southern blot analysis. (G) Functional assessment of epitope-tagged CK2 components. The wild-type and mutant strains – *cka1*Δ::*CKA1-4xFLAG-NEO* (YSB7784), *ckb1*Δ::*CKB1-4xFLAG-NEO* (YSB7812), *ckb1*Δ::*CKB1-6xHA-NEO* (YSB8251), *ckb2*Δ::*CKB2-6xHA-NEO* (YSB7846), *cka1*Δ::*CKA1-4xFLAG-NEO CKB1*:*6xHA-HYG* (YSB8081), *cka1*Δ::*CKA1-4xFLAG-NEO CKB2*:*6xHA-HYG* (YSB8179), and *ckb1*Δ::*CKB1-4xFLAG-NEO CKB2*:*6xHA-HYG* (YSB8082) – were cultured overnight at 30°C in YPD broth, serially diluted (1 to 10^4), and spotted onto YPD solid medium supplemented with specific stress inducers. (H) Structural predictions of CK2 regulatory subunits in *H. sapiens*, *C. neoformans*, *S. cerevisiae*, *C. albicans*, and *S. pombe*. The three-dimensional structures for *H. sapiens* CK2β, *C. neoformans* proteins Ckb1 and Ckb2, *S. cerevisiae* proteins Ckb1 and Ckb2, *C. albicans* proteins Ckb1 and Ckb2, and *S. pombe* proteins Ckb1 and Ckb were computationally predicted using AlphaFold2 (ColabFlod v1.5.2).
