# Supplementary material for: Casein kinase 2 complex: a central regulator of multiple pathobiological signaling pathways in Cryptococcus neoformans
Source: mBio. 2024 Jan 9;15(2):e03275-23. doi: 10.1128/mbio.03275-23 (PMC10865844; doi:10.1128/mbio.03275-23)
Supplement: Fig. S4 — Generation and validation of mCherry-tagged CK2 strains. [file mbio.03275-23-s0007.pdf]

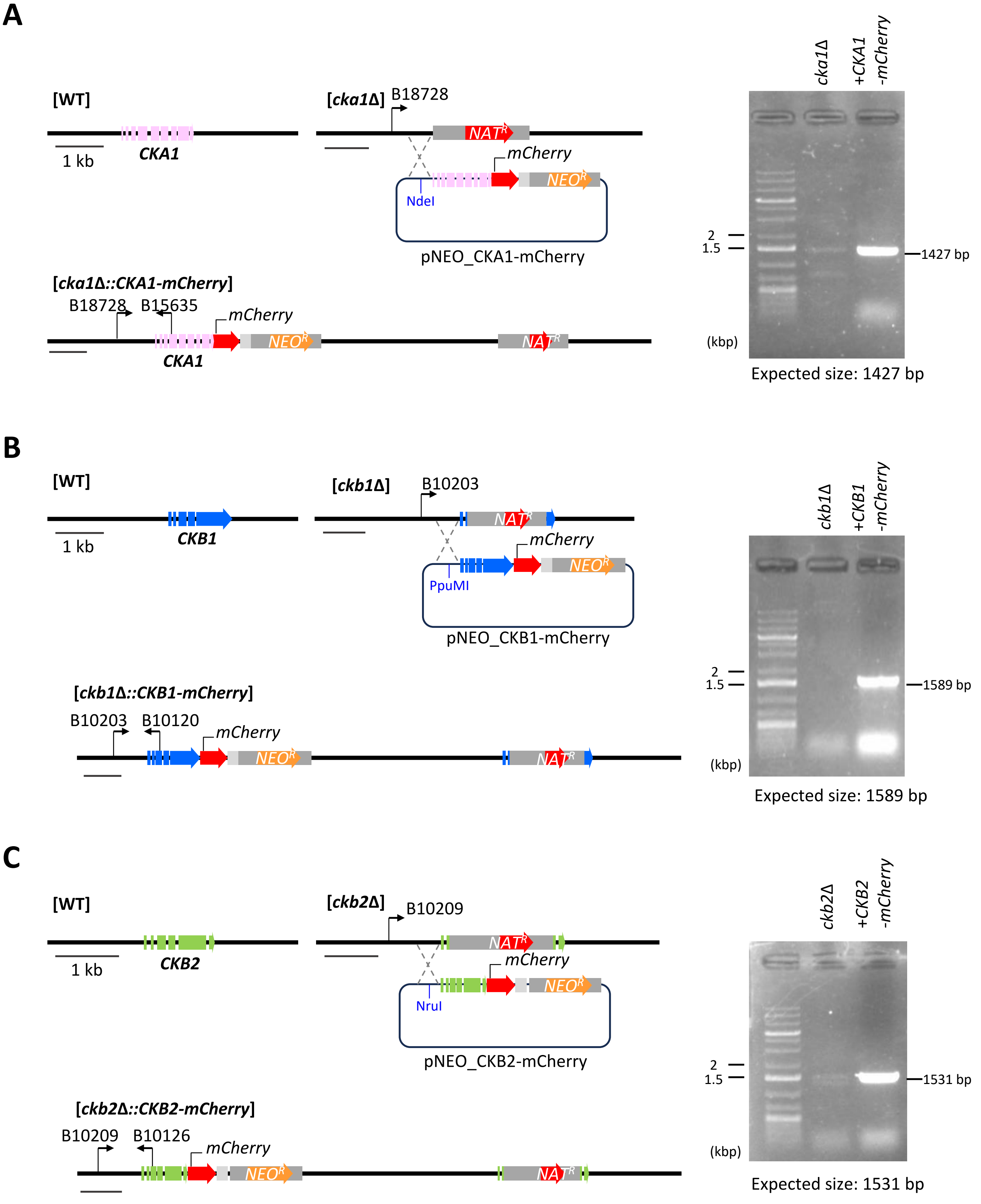
A
[WT]
1 kb
CKA1
[cka1Δ]
B18728
NAT^R
mCherry
NEO^R
NdeI
pNEO_CKA1-mCherry
[cka1Δ::CKA1-mCherry]
B18728
B15635
mCherry
NEO^R
CKA1
NAT^R
cka1Δ
+CKA1 -mCherry
2
1.5
(kbp)
1427 bp
Expected size: 1427 bp
B
[WT]
1 kb
CKB1
[ckb1Δ]
B10203
NAT^R
mCherry
NEO^R
PpuMI
pNEO_CKB1-mCherry
[ckb1Δ::CKB1-mCherry]
B10203
B10120
mCherry
NEO^R
NAT^R
ckb1Δ
+CKB1 -mCherry
2
1.5
(kbp)
1589 bp
Expected size: 1589 bp
C
[WT]
1 kb
CKB2
[ckb2Δ]
B10209
NAT^R
mCherry
NEO^R
NruI
pNEO_CKB2-mCherry
[ckb2Δ::CKB2-mCherry]
B10209
B10126
mCherry
NEO^R
NAT^R
ckb2Δ
+CKB2 -mCherry
2
1.5
(kbp)
1531 bp
Expected size: 1531 bp

D

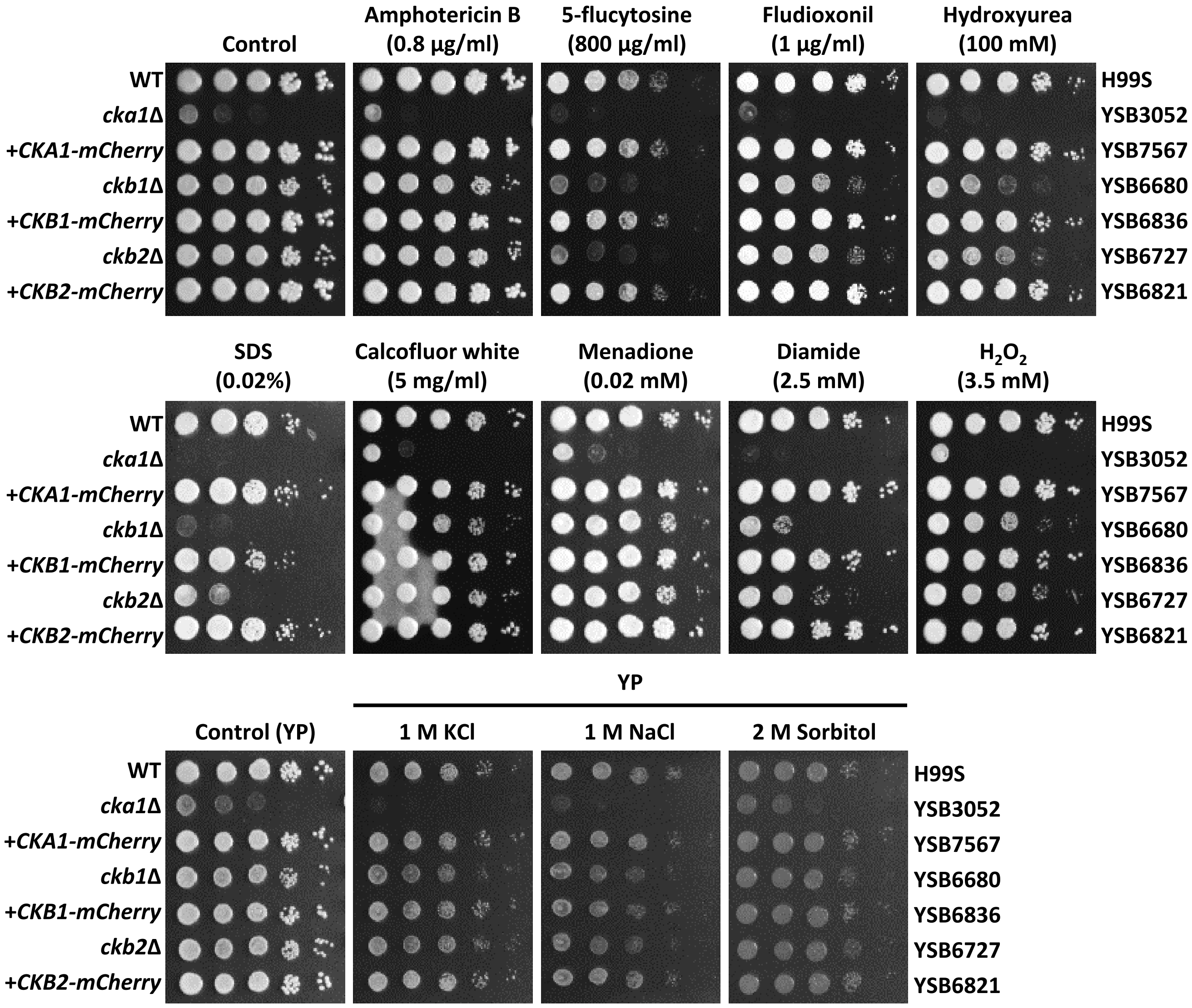

FIG S4. Generation and validation of mCherry-tagged CK2 strains. To generate mCherry-tagged CK2 components, the DNA sequences encompassing both the promoter and ORF for *CKA1* (A), *CKB1* (B), *CKB2* (C) was subcloned into the pNEO_mCherry vector. After linearizing the resultant plasmids with specific restriction enzymes, they were introduced into the corresponding deletion mutants – *cka1*Δ (YSB3052), *ckb1*Δ (YSB6680), and *ckb2*Δ (YSB6727) – via biolistic transformation. The successful targeted integration of these constructs was verified through diagnostic PCR. (D) Functional assessment of mCherry-tagged CK2 strains. To ascertain the functionality of the mCherry-tagged CK2 components, the wild-type and CK2 mutant strains – *cka1*Δ (YSB3052), *cka1*Δ::*CKA1-mCherry* (YSB7567), *ckb1*Δ (YSB6680), *ckb1*Δ::*CKB1-mCherry* (YSB6836), *ckb2*Δ (YSB6727), and *ckb2*Δ::*CKB2-mCherry* (YSB6821) – were cultured overnight at 30°C in YPD broth, serially diluted (1 to 10^4), and then spotted onto YPD plates containing specific stress inducers.
