# Supplementary material for: Casein kinase 2 complex: a central regulator of multiple pathobiological signaling pathways in Cryptococcus neoformans
Source: mBio. 2024 Jan 9;15(2):e03275-23. doi: 10.1128/mbio.03275-23 (PMC10865844; doi:10.1128/mbio.03275-23)
Supplement: Fig. S5 — The role of CK2 in stress adaptation and virulence in C. neoformans. [file mbio.03275-23-s0008.pdf]

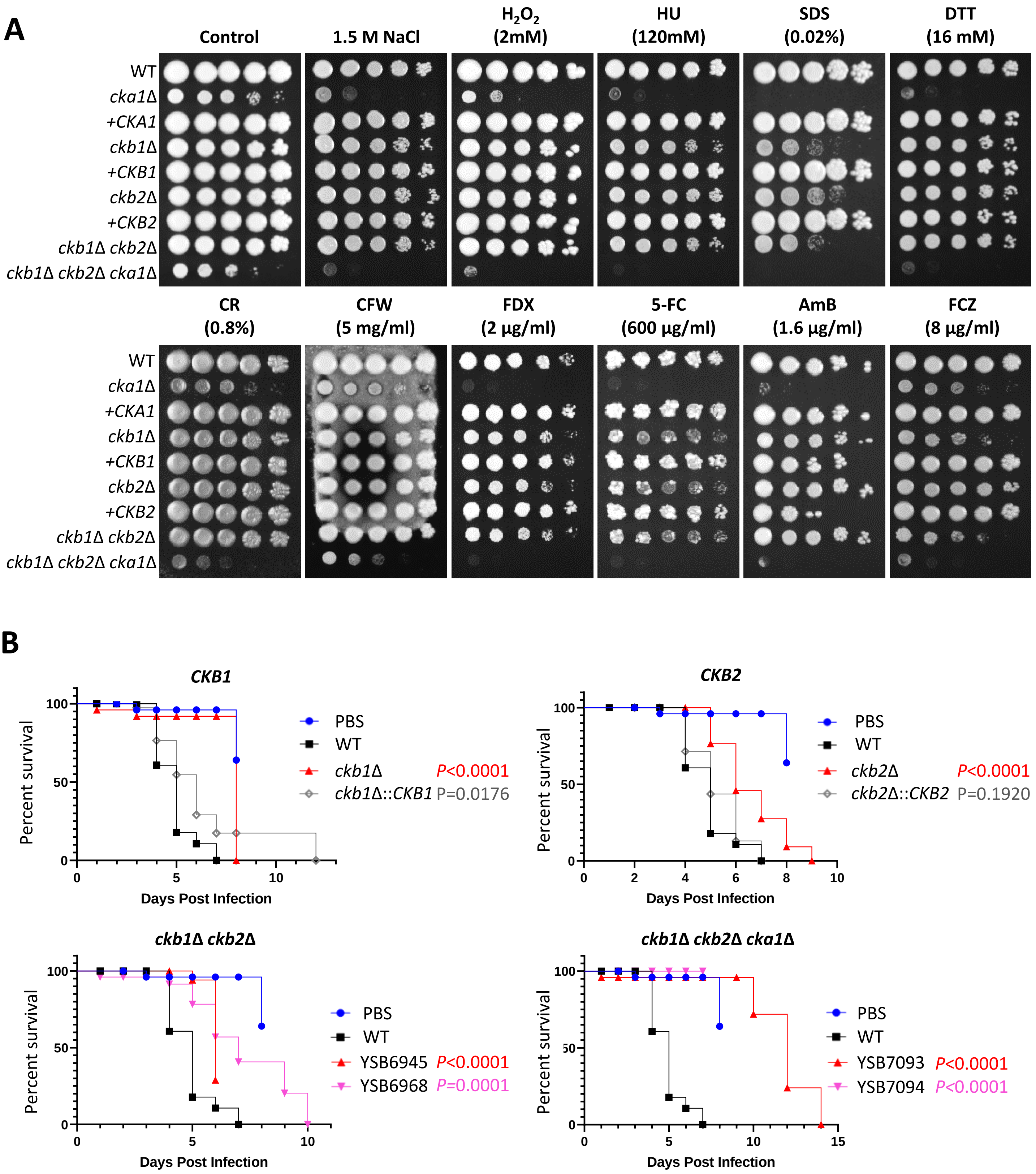

FIG S5. The role of CK2 in stress adaptation and virulence in *C. neoformans*. (A) Stress susceptibility assay. Stress response and adaptation of wild-type (H99S), *cka1*Δ (YSB3052), *cka1*Δ::*CKA1* (YSB6746), *ckb1*Δ (YSB6680), *ckb1*Δ::*CKB1* (YSB6840), *ckb2*Δ (YSB6727), *ckb2*Δ::*CKB2* (YSB6826), *ckb1*Δ *ckb2*Δ (YSB6945), and *ckb1*Δ *ckb2*Δ *cka1*Δ (YSB7093) were assessed. Strains were cultured overnight at 30°C in liquid YPD, serially diluted (1 to 10^4), and spotted onto YPD solid medium containing the specific stress inducers. The plates were incubated at 30°C and photographed on day 6. (B) An insect-killing assay was conducted to evaluate the virulence of CK2 mutants. The statistical differences in virulence between the wild-type strain (H99S) and each mutant strain – *ckb1*Δ (YSB6680), *ckb1*Δ::*CKB1* (YSB6840), *ckb2*Δ (YSB6727), *ckb2*Δ::*CKB2* (YSB6826), *ckb1*Δ *ckb2*Δ (YSB6945 and YSB6968), and *ckb1*Δ *ckb2*Δ *cka1*Δ (YSB7093 and YSB7094) were assessed using the log-rank (Mantel–Cox) test to determine the *P* values.
