# Supplementary material for: Casein kinase 2 complex: a central regulator of multiple pathobiological signaling pathways in Cryptococcus neoformans
Source: mBio. 2024 Jan 9;15(2):e03275-23. doi: 10.1128/mbio.03275-23 (PMC10865844; doi:10.1128/mbio.03275-23)
Supplement: Fig. S6 — Transcriptomic analysis and detailed proteomic and phosphoproteomic analysis overview. [file mbio.03275-23-s0009.pdf]

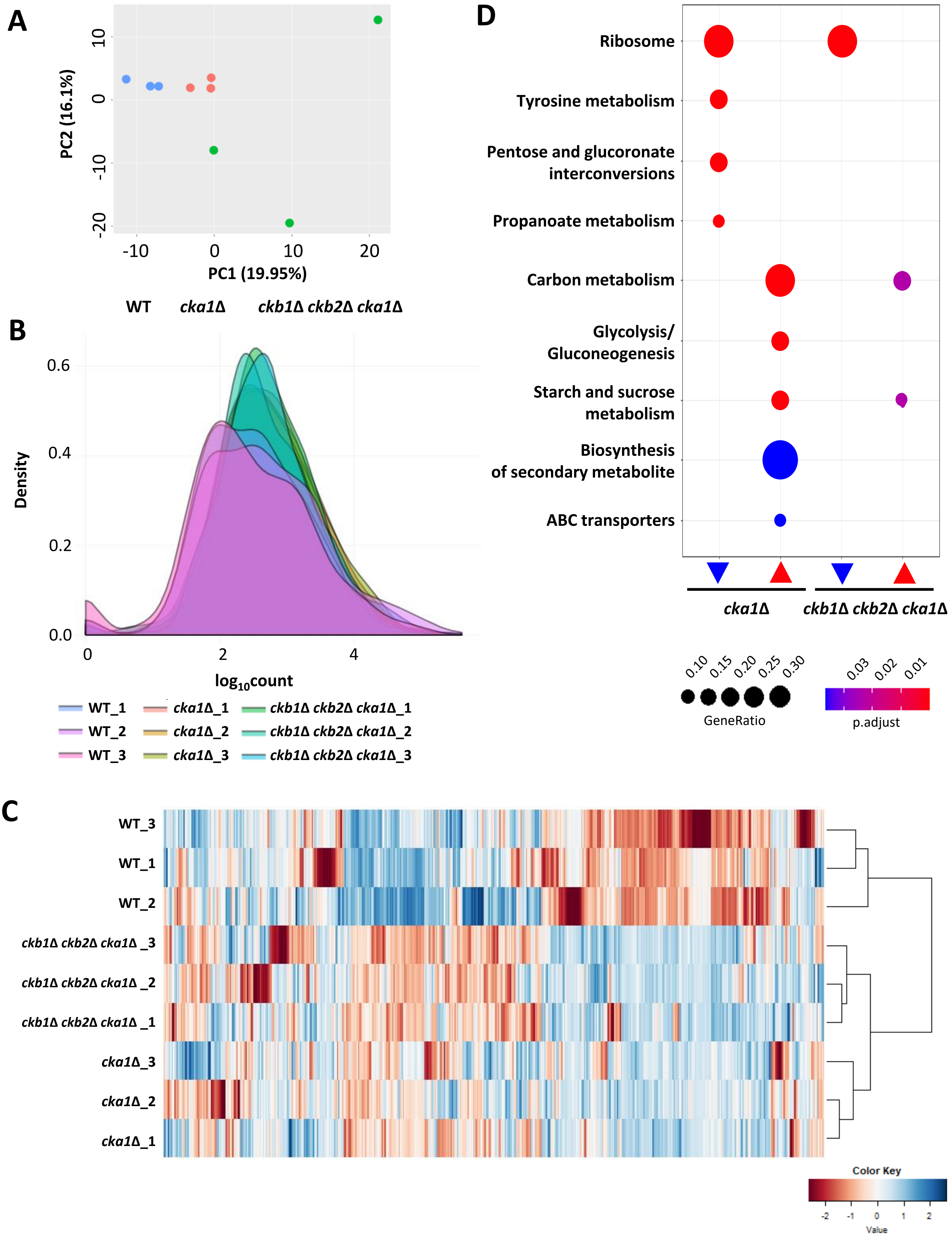

A
PC2 (16.1%)
PC1 (19.95%)
10
0
-10
-20
-10
0
10
20
WT
cka1Δ
ckb1Δ ckb2Δ cka1Δ
B
Density
0.6
0.4
0.2
0.0
0
2
4
log10count
WT_1
WT_2
WT_3
cka1Δ_1
cka1Δ_2
cka1Δ_3
ckb1Δ ckb2Δ cka1Δ_1
ckb1Δ ckb2Δ cka1Δ_2
ckb1Δ ckb2Δ cka1Δ_3
C
WT_3
WT_1
WT_2
ckb1Δ ckb2Δ cka1Δ _3
ckb1Δ ckb2Δ cka1Δ _2
ckb1Δ ckb2Δ cka1Δ _1
cka1Δ_3
cka1Δ_2
cka1Δ_1
Color Key
-2
-1
0
1
2
Value
D
Ribosome
Tyrosine metabolism
Pentose and glucoronate interconversions
Propanoate metabolism
Carbon metabolism
Glycolysis/ Gluconeogenesis
Starch and sucrose metabolism
Biosynthesis of secondary metabolite
ABC transporters
cka1Δ
ckb1Δ ckb2Δ cka1Δ
0.10
0.15
0.20
0.25
0.30
GeneRatio
0.03
0.02
0.01
p.adjust

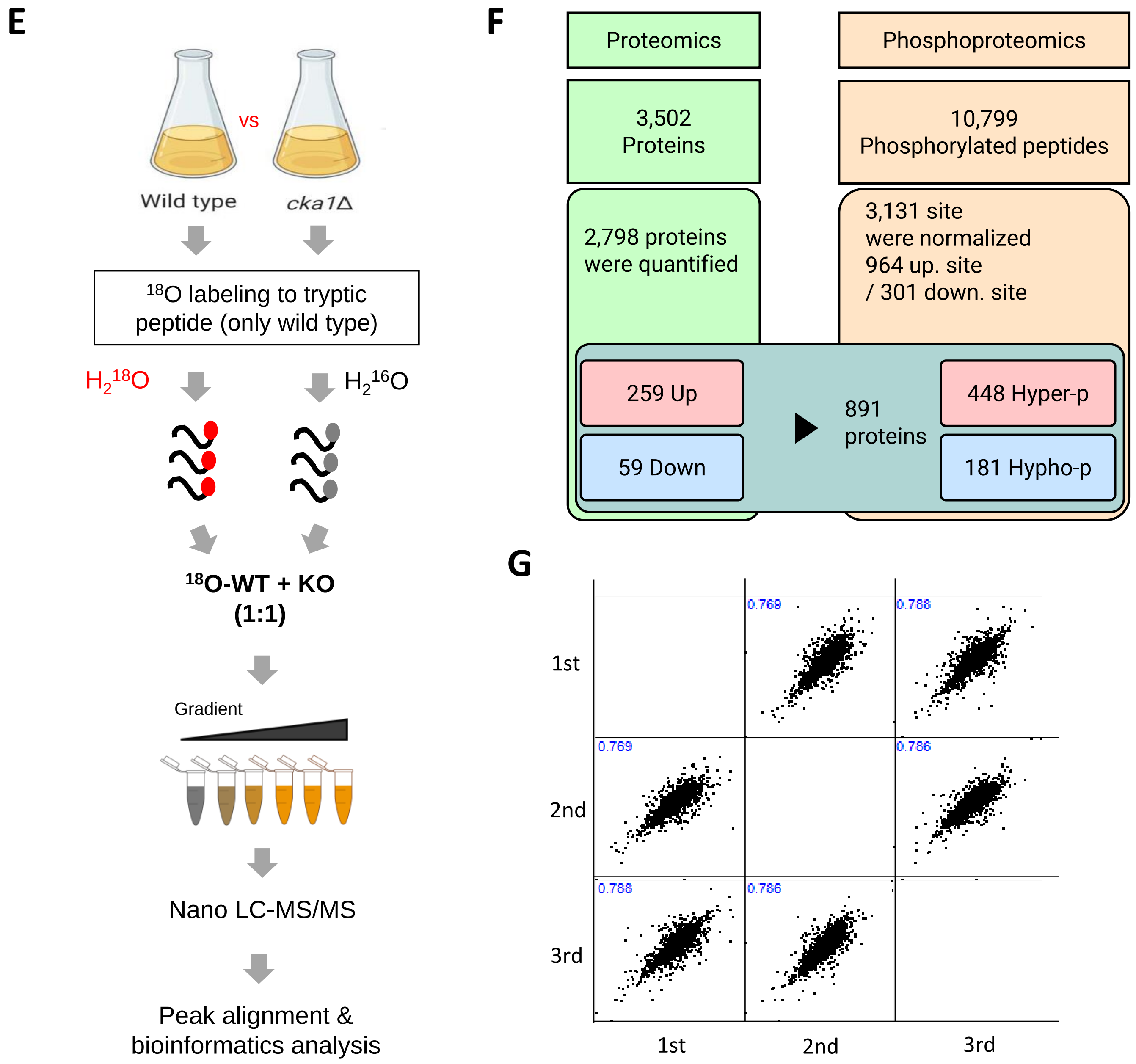

FIG S6. Transcriptomic analysis of *cka1*Δ and *ckb1*Δ *ckb2*Δ *cka1*Δ strains and detailed proteomic and phosphoproteomic analysis overview. (A-C) Transcriptome data quality assessment. Using debrowser (v1.22.4) with the 'startdebrowser' command, the quality of the transcriptomic datasets was evaluated. A principal component analysis (PCA) plot (A) provides an overview of sample clustering. The read count density plot (B) offers insights into sequencing depth. The heatmap plot (C) indicates sample-to-sample similarity, underscoring that the three biological replicates group together coherently. (D) KEGG pathway analysis. Comparative pathway analysis, based on the Kyoto Encyclopedia of Genes and Genomes (KEGG), was performed for the *cka1*Δ and *ckb1*Δ *ckb2*Δ *cka1*Δ mutants in comparison to wild-type. These KEGG analyses were executed using enrichKEGG analysis in Clusterprofiler (https://www.genome.jp/kegg, Clusterprofiler 3.6.0). Within the graphical representation, circle sizes correlate with gene ratios. The color gradient, transitioning from blue to red, symbolizes a range from high to low P_{adjust} values, respectively. (E) Quantitative proteomic analysis workflow. Proteins were first digested with Trypsin, subsequently labeled with ^{18}O, and then subjected to analysis using nano-LC coupled with LTQ-Velos orbitrap mass spectrometry. (F) A graphic representation showing the count of proteins distinctly identified in both wild-type and *cka1*Δ groups by proteomic and phosphoproteomic analyses. (G) Reproducibility among triplicate samples. Pearson's correlation analysis showcases the consistency and reproducibility of the data among triplicate samples.
