# Supplementary material for: Casein kinase 2 complex: a central regulator of multiple pathobiological signaling pathways in Cryptococcus neoformans
Source: mBio. 2024 Jan 9;15(2):e03275-23. doi: 10.1128/mbio.03275-23 (PMC10865844; doi:10.1128/mbio.03275-23)
Supplement: Fig. S7 — Generation and characterization of the cka1 mutant expressing Crz1-mCherry. [file mbio.03275-23-s0010.pdf]

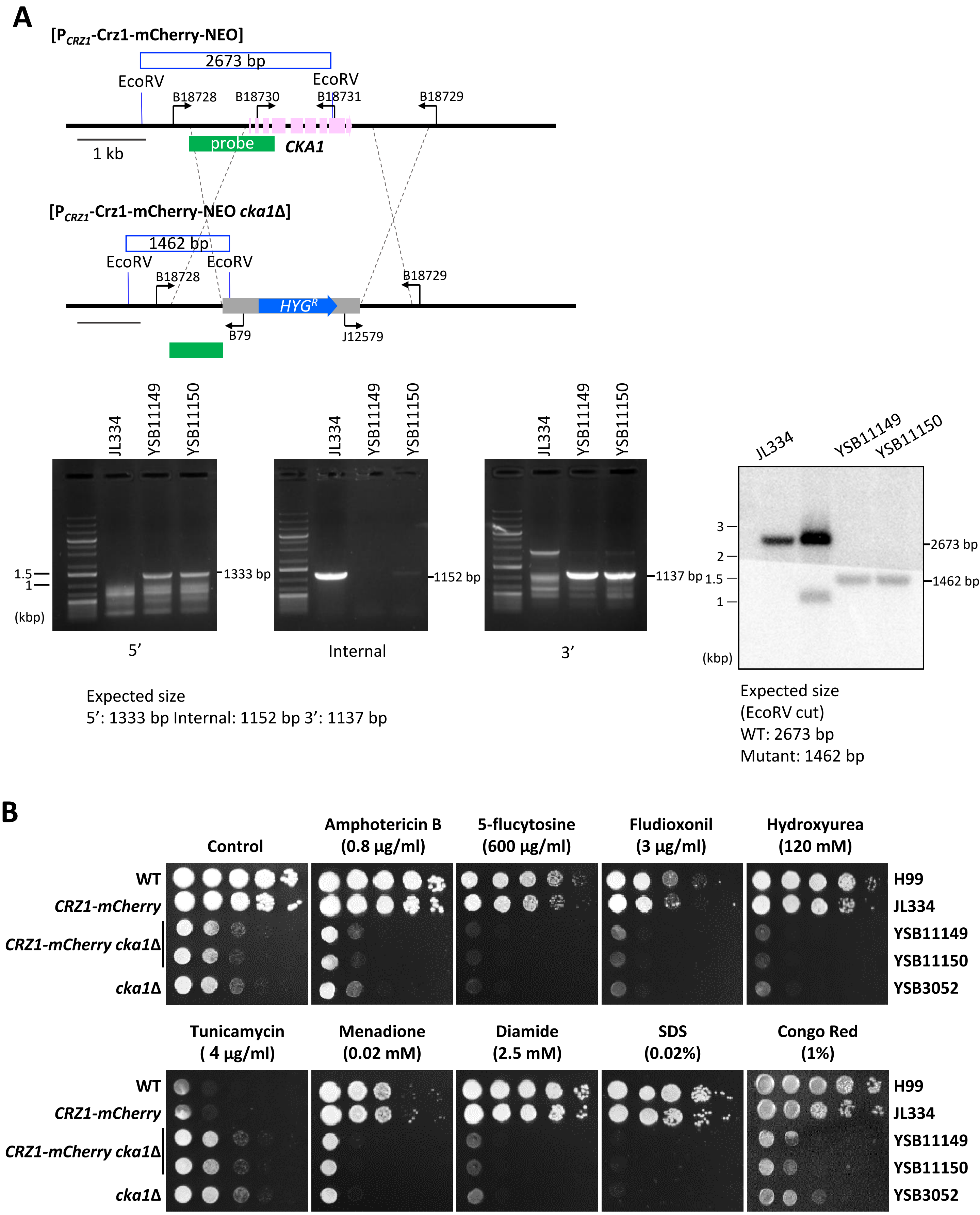


**FIG S7. Generation and characterization of the *cka1*Δ mutant expressing Crz1-mCherry.** (A) Genotype analysis of the *cka1*Δ mutants. The top panel shows the gene disruption strategy depicting the replacement of the *CKA1* gene with the *NAT*-selection marker. The bottom right panel presents diagnostic PCR results confirming the recombination event at the 5′-end and 3′-end, alongside the internal deletion of the *CKA1* gene. For Southern blot analysis, genomic DNA from the *CRZ1*:*mCherry-NEO* strain (JL334) and *CRZ1*:*mCherry-NEO cka1*Δ mutants (YSB11149 and YSB11150) underwent EcoRV digestion. (B) Phenotypic analysis of *cka1*Δ mutants. Each strain – wild-type (H99S), *CRZ1*:*mCherry-NEO* (JL334), *CRZ1*:*mCherry-NEO cka1*Δ (YSB11149 and YSB11150), and *cka1*Δ (YSB3052)) – was cultured overnight in YPD broth at 30°C, serially diluted (1 to $10^4$), and spotted onto YPD plates containing the following stress inducers: amphotericin B, 5-fluorocytosine, fludioxonil, hydroxyurea, tunicamycin, menadione, diamide, sodium dodecyl sulfate (SDS), or Congo red. The plates were further incubated at 30°C for 4 days.
